# A novel mutation in FDX2 provides insights into the pathogenesis of MEOAL mitochondrial neuromuscular disease

**DOI:** 10.1038/s41419-025-08323-3

**Published:** 2025-12-10

**Authors:** Davide Doni, Deborah Grifagni, Federica Cavion, Bianca Buchignani, Roberta Battini, Elisa Baschiera, Maria Andrea Desbats, Rosa Pasquariello, Giuseppina Covello, Eva De Pascale, Alice Boarolo, Ilaria Cestonaro, Denis Badocco, Paolo Pastore, Geppo Sartori, Oliver Stehling, Roland Lill, Filippo M. Santorelli, Leonardo Salviati, Simone Ciofi-Baffoni, Paola Costantini

**Affiliations:** 1 Department of Biology, University of Padova, 35121 Padova, Italy; 2 Magnetic Resonance Center (CERM), University of Florence, 50019 Sesto Fiorentino, Italy; 3 Department of Chemistry ’Ugo Schiff‘ (DICUS), University of Florence, 50019 Sesto Fiorentino, Italy; 4 Department of Developmental Neuroscience, IRCCS Fondazione Stella Maris, 56018 Calambrone Pisa, Italy; 5 Department of Translational Research and of New Surgical and Medical Technologies, University of Pisa, Pisa, Italy; 6 Department of Clinical and Experimental Medicine, University of Pisa, Pisa, Italy; 7 Clinical Genetics Unit, Department of Women’s and Children Health, University of Padova, 35128 Padova, Italy; 8 Istituto di Ricerca Pediatrica (IRP) Città della Speranza, 35127 Padova, Italy; 9 Department of Chemical Sciences, University of Padova, 35131 Padova, Italy; 10 Department of Biomedical Sciences, University of Padova, 35121 Padova, Italy; 11 Institut für Zytobiologie im Zentrum für Synthetische Mikrobiologie SynMikro, Philipps-Universität Marburg, Marburg, Germany; 12 Neurobiology and Molecular Medicine Unit, IRCCS Fondazione Stella Maris, 56128 Calambrone Pisa, Italy

**Keywords:** Mechanisms of disease, Neuromuscular disease

## Abstract

Episodic mitochondrial myopathy with or without optic atrophy and reversible leukoencephalopathy (MEOAL) is a rare autosomal recessive neuromuscular disorder characterized by childhood onset of progressive muscle weakness and exercise intolerance. It is caused by mutations in the *FDX2* gene, encoding the mitochondrial protein ferredoxin 2 (FDX2), a central component of the cellular FeS protein biogenesis. To date there are gaps in our understanding of how FDX2 mutations impact mitochondrial pathophysiology in MEOAL patients. In this work we report a multidisciplinary study of a pediatric patient with a diagnosis of neuromuscular disorder, with multiorgan involvement, associated with a novel homozygous mutation in *FDX2*, i.e., c.200+4 A > G. We found that: (i) the mutation alters the splicing of the gene transcript, giving rise to a mutant protein in which 19 N-terminal residues encoded by exon 2 are replaced by 21 different amino acids; (ii) patient’s cells have low levels of FDX2; (iii) the mutant FDX2 likely retains its functional integrity, as can be inferred by the absence of significant structural or backbone dynamic differences relative to the wild type protein; (iv) cultured patient’s cells show impaired mitochondrial respiration, defects in many FeS proteins, and enhanced mitochondrial iron accumulation; (v) the levels of the mitochondrial SOD2 are significantly diminished in patient’s cells and may contribute to weak ROS production. Collectively, the results show that the *FDX2* mutation leads to a severe decrease of FDX2 protein, resulting in a primary mild cellular FeS protein assembly defect and in the secondary consequences mentioned above, that together may explain the pathogenesis of this MEOAL case.

## Introduction

Episodic mitochondrial myopathy with or without optic atrophy and reversible leukoencephalopathy (MEOAL, OMIM number: 251900) is a rare, orphan autosomal recessive neuromuscular disorder characterized by onset in childhood or adolescence of recurrent episodes of cramps, myalgia, and muscle weakness, often triggered by exercise, infection or low temperatures. MEOAL is caused by mutations in the *FDX2* gene, encoding the mitochondrial protein ferredoxin 2, ubiquitously expressed in human tissues [1–6]. To date, only eleven MEOAL patients have been reported by the medical literature. Different mutations of FDX2 lead either to a relatively mild skeletal phenotype or to a complex multisystem neurological/neuromuscular syndrome. The first case of mitochondrial myopathy associated with FDX2 was described in 2014 in a 15-year-old patient with recurrent myoglobinuria, lactic acidosis, and slowly progressive muscle weakness due to a homozygous mutation affecting the translation start codon of *FDX2* (c.1 A > T, p.Met1Leu) and resulting in a severe decrease in FDX2 protein [1]. The same mutation has been more recently found in four patients with a similar muscular phenotype, in most cases associated with severe rhabdomyolysis [2–5]. In 2018, the homozygous missense mutation c.431 C > T (p.P144L) in *FDX2* was described in six patients from two unrelated families with autosomal recessive inheritance of a complex neurological phenotype involving early onset optic atrophy followed in the first or second decade of life by progressive myopathy, recurrent episodes of cramps, myalgia, muscle weakness, and axonal polyneuropathy [6]. Muscle biopsies from MEOAL patients carrying this mutation showed very low amounts of FDX2 protein, while mRNA levels were unaffected, compared to control samples.

A challenge in elucidating the pathological picture of MEOAL disease stems from the restricted knowledge of the physiological consequences of FDX2 deficiency in cellular dysfunction. FDX2 was found to work as an electron transfer protein in the mitochondrial biosynthesis of iron-sulfur (FeS) clusters and, indirectly, heme prosthetic groups of the respiratory complexes and many other enzymes [7–13]. Electron transfer by human FDX2 is required for assembling both [2Fe-2S] and [4Fe-4S] clusters [7–12, 14, 15]. Mitochondrial FeS cluster assembly also supports cytosolic-nuclear FeS cluster biogenesis by the CIA machinery [16–18]. Thus, mitochondrial FDX2 is a critical element for many cellular physiological pathways involving FeS proteins. Consequently, the deficiency of FDX2 is associated with impaired mitochondrial energy metabolism including defective organellar respiration as well as biosynthesis of heme B and lipoic acid moieties via the failure of the maturation of [2Fe-2S] ferrochelatase and [4Fe-4S] lipoyl synthase, respectively [12, 19]. Hence, a common biochemical hallmark of cells from the few MEOAL patients described to date is the decrease of the enzymatic activities of the respiratory complexes, which rely on FeS clusters (I, II, and III) and heme (IV) as electrons shuttles [1, 2]. Therefore, defects in the biogenesis of the FeS proteins due to FDX2 mutations are expected to contribute to the MEOAL pathology onset and progression by impairing crucial mitochondrial functions. Currently, no specific treatments are available for MEOAL patients, and their medical care is essentially aimed at mitigating the symptoms.

In this work, we report a multidisciplinary study of a pediatric patient with a diagnosis of neuromuscular disorder associated with a novel homozygous mutation in *FDX2*, i.e., c.200+4 A > G. We combined the description of the patient’s clinical phenotype with molecular, chemical/biochemical, and mitochondrial physiology analyses on whole cells and structural studies on the mutant FDX2 protein, to gain new clues on the pathophysiological mechanisms leading to MEOAL disease.

## Results

### Clinical findings

**Fig. 1:**
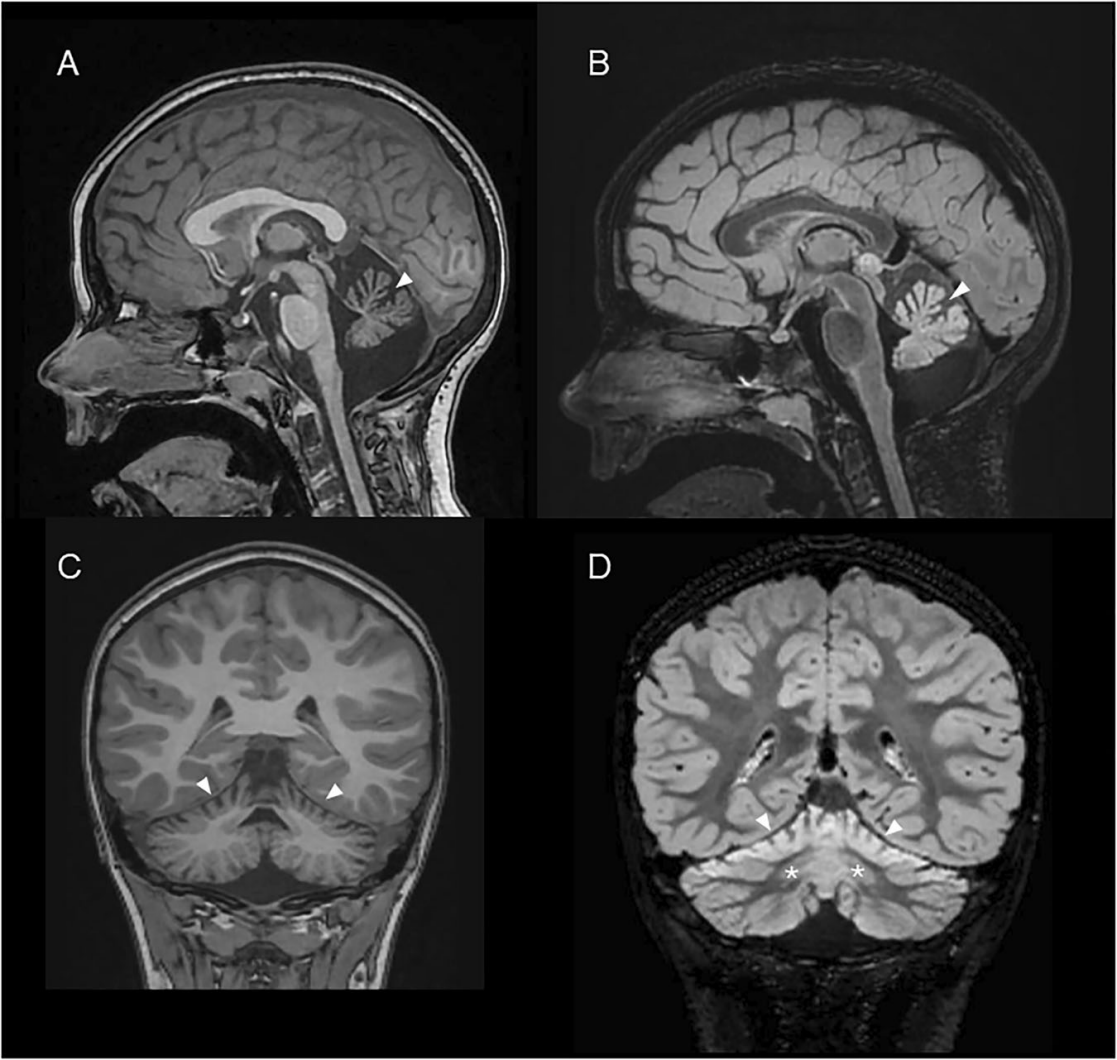
Brain MRI of MEOAL patient. Sagittal and coronal t1 MPRAGE (**A**, **C**) sagittal and coronal FLAIR3D brain images (**B**, **D**). Thinning of the superior cerebellar folia and vermis with cortical and subcortical hyperintensity of cerebellar hemisphere and vermis (arrows). Mild bilaterally hyperintensity of nucleus dentatus (*).

The patient, now 9 years old, was born at term by cesarean with suspected suffering at birth. She was mildly hypotonic, presenting with poor sucking and had some difficulties in gaining weight. No delays on her motor milestones were noticed until the age of 9 months, when high temperature, weakness, and difficulty to crawl was observed. After one more month she progressed in her motor abilities. However, she did not acquire independent walking without aid. Her language milestones were on time. When she was first referred to the hospital her clinical picture was characterized by severe ataxic signs (trunk and head ballottement, dysarthria, dysmetria, intentional tremor, and nystagmus), hyposthenia, and pyramidal signs (clonus, positive Babinski reflex, and increase of patellar reflexes). Moreover, upon clinical observation she presented inverted nipples and an abnormal accumulation of fat. A psychological evaluation was conducted and revealed a normal intelligence quotient; no behavioral issues were observed. A first MRI at 2 years of age showed cerebellar hypoplasia which appeared as a progressive cerebellar atrophy in subsequent MRIs, performed at 4, 5, 6 (Fig. 1) and 8 years of age. In particular, the atrophy involved both the vermis and hemispheric expression associated with shaded hyperintensity T2/FLAIR of the supero-lateral cortex of the cerebellar and superior vermis hemispheres. No seizures or epileptic signs were highlighted at an electroencephalogram. Blood samples showed a mild increase in lactic acid, while no other metabolic parameters were abnormal. Her clinical picture has changed over time. The girl has progressively become more hypostenic, presented more fatigue during walking with support and showed a gain of weight also due to an increase in appetite. Moreover, a progressive ocular involvement with foveal hypoplasia has been found overtime. Studies carried out on her blood continue to show no anemia or thyroid dysfunction. Moreover, no acute events such as rhabdomyolysis have occurred. Her nerve conductions have shown no progression over time, nor has an MRI shown further progression.

Based on the clinical picture and symptoms, an involvement of mitochondria was hypothesized; the patient started a multivitamin therapy (cocktail of riboflavin, thiamin and coenzyme Q) when she was three years old.

### Genetic analysis

**Fig. 2:**
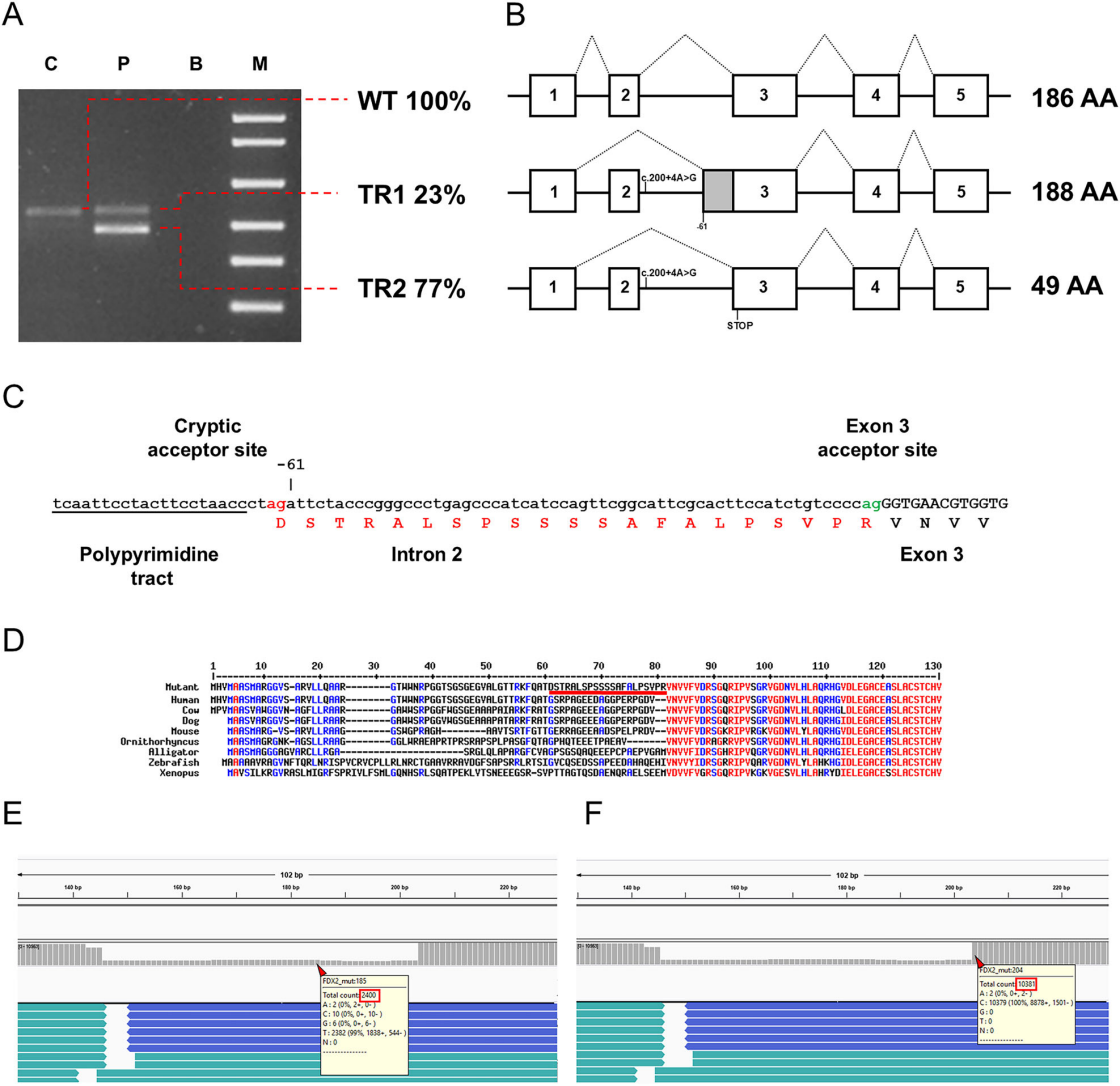
Effect of the c.200+4 A > G mutation in *FDX2* transcription. **A** RT-PCR products separated on a 1% agarose gel. C Control, P Patient, B Blank, M = 1KB DNA Ladder. Relative abundance of individual transcripts was estimated using densitometric analysis. **B** Structure of the 3 different transcripts (WT, TR1, and TR2)**. C** Structure of the region encompassing the 3’ of intron 2 and the 5’ of exon 3. Underlined the polypyrimidine tract preceding the cryptic splice site. Below is the predicted translation (in red the 21 abnormal amino acids). **D** Alignments of FDX2 proteins from different vertebrate species. Underlined in red the 21 abnormal amino acids in the mutant protein. **E**, **F** FASTQ files from the patient aligned with the sequence of TR1 and visualized with IGV software. In the red boxes the number of reads mapping to the intronic region (unique to TR1) (**E**) and those mapping to exon 3 (total transcripts) (**F**).

An NGS molecular analysis of patient’s genomic DNA captured by a panel of mitochondrial-related genes was performed. The screening revealed a likely pathogenic homozygous variant in *FDX2* gene, located at position +4 from the intron 2 donor site (c.200+4 A > G). The segregation analysis showed the presence of the heterozygous variant in both parents. To confirm the potential pathogenic role of the mutation in altering splicing at intron 2, we analyzed the gene transcripts. Total RNAs were extracted from cultured primary skin fibroblasts from the patient and from an age-matched healthy control and retrotranscribed as described in Materials and Methods. Amplification of the *FDX2* cDNA yielded a single band of the expected size in the control sample (WT), while the patient sample yielded two products (Fig. 2A and B), one with a size similar to the wild type (TR1—23% of the transcripts) and a lower band (TR2—77% of the transcripts). Sanger sequencing of individual bands excised from the gel confirmed that the wild type fragment corresponded to the correctly spliced *FDX2* cDNA, and revealed that TR2 lacked exon 2, whereas TR1 did not entirely correspond to the normal *FDX2* transcript. Rather, TR1 was a transcript also lacking exon 2 but, however, retaining 61 nucleotides from the end of intron 2, due to the activation of an intronic cryptic splice acceptor site which is preceded by a bona-fide polypyrimidine tract (Fig. 2C). *FDX2* exon 2 is comprised of 55 nucleotides, therefore in TR2 the reading frame is shifted causing the formation of a premature stop codon at amino acid position 50 (Fig. 2B). In TR1 the reading frame is preserved and the 19 amino acids encoded by exon 2 are replaced by 21 amino acids encoded by intron 2, while the rest of the trascript is normal (Fig. 2B). The resulting protein, hereafter referred to as mutant FDX2, is two amino acids longer than the wild type protein but retains the normal N-terminus including the predicted mitochondrial targeting sequence (MTS). The mutant domain encoded by the intronic sequence is located downstream of the MTS and corresponds to a poorly conserved N-terminal region of mature FDX2 (Fig. 2D).

To check whether low levels of normal transcripts, which could have been missed by Sanger sequencing, were indeed present in the patient, aliquots of control and patient-derived PCR products were also sequenced using an NGS amplicon resequencing method. Again, the transcript found in the control did not show any abnormalities. When the FASTQ file of the patient sample was aligned to the sequence of the wild type transcript, there were no reads mapping to exon 2. Conversely, when we aligned the FASTQ file to the sequence of TR1, coverage for the retained intronic region (corresponding to TR1) was around 2400x (Fig. 2E), while coverage for the exon 3 region (corresponding to the total transcripts) was approximately 10,380x (Fig. 2F). Interestingly, the sequencing data indicate that the relative amounts of TR1 and TR2 are 23% and 77%, confirming the densitometric analysis.

### Structural properties of mutant FDX2

We addressed whether the different N-terminal segment of mutant FDX2 with respect to that of the wild type protein affects the cluster binding as well as redox and structural/backbone dynamic properties of FDX2. To this end, we generated two recombinant constructs corresponding either to the mature wild type or to the mutant FDX2, each devoid of the MTS. The MTS cleavage site motif of wild type protein is predicted to be constituted by xRx(F/L/I)xx(S/T/G)xxxx ↓ , according to literature [20] and UNIPROT database, and it is conserved in the sequence of the mutant FDX2 (Fig. 2D). Thus, we can predict that the mature form of the mutant FDX2 in mitochondria of MEOAL patient’s cells is generated by the same cut occurring in wild type FDX2. We therefore produced in *Escherichia coli* a wild type FDX2 spanning from Ala53 to His183 and a mutant FDX2 spanning from Ala53 to His185. To further explore the impact of the alteration of the N-terminal portion of FDX2, we also generated a recombinant protein spanning from Asp66 to His183, which has been previously functionally and structurally characterized [21], and where thirteen N-terminal residues following the chopped MTS were removed (FDX2^66-183^) in addition (see Supplementary Fig. 1 for the sequences of the three recombinant proteins).

**Fig. 3:**
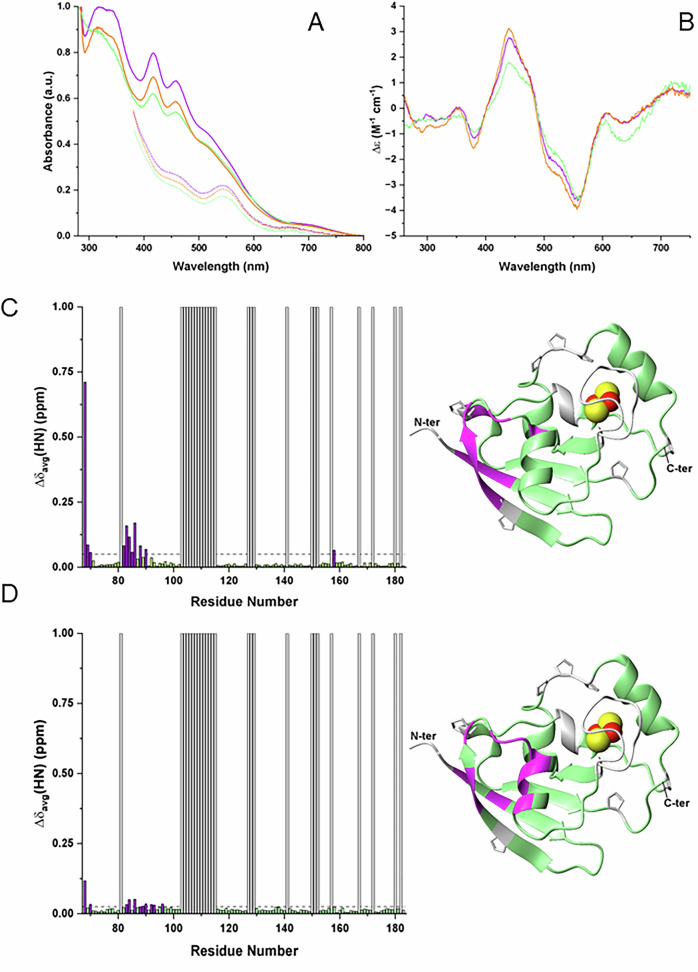
Comparative spectroscopic analysis of wild type [2Fe-2S] FDX2, [2Fe-2S] mutant FDX2 and [2Fe-2S] FDX2^66-183^ and analysis of the interaction between the N-terminal segments of mutant and wild type FDX2 proteins and the globular domain of [2Fe-2S] FDX2. The UV/visible (**A**) and UV/visible-CD (**B**) spectra recorded at 298 K on the [2Fe-2S]^2+^ mutant FDX2 (purple), [2Fe-2S]^2+^ wild type FDX2 (green) and [2Fe-2S]^2+^ FDX2^66-183^ (orange). UV/visible spectra of the three dithionite-reduced [2Fe-2S]^+^ FDX2 proteins (dotted lines) are also reported. The latter spectra were cut at 380 nm as a consequence of the presence at 314 of the strong absorption of dithionite in excess. Δε values of UV/visible-CD spectra are based on [2Fe-2S] concentration. Buffer conditions: 30 mM HEPES, 150 mM NaCl at pH 7.5. **C** On the left, backbone weighted average chemical shift differences (Δδ_avg_(HN) for residues 68-183) between [2Fe-2S]^2+^ mutant FDX2 and [2Fe-2S]^2+^ FDX2^66-183^ are reported. A chemical shift threshold value of 0.05 ppm, indicated as a dashed line, was estimated to define the significant chemical shift differences. The white bars indicate proline and unassigned NHs. Purple bars indicate the residues with chemical shift changes larger than the threshold value. On the right, the chemical shift changes, which are larger than the threshold value of 0.05 ppm, are mapped in purple on the backbone of the crystal structure of [2Fe-2S] wild type FDX2 (residues 66-171). The backbone of prolines, of unassigned residues and of the three N-terminal residues (residues 65-67), the latter having different kinds of amino acids comparing the sequences of [2Fe-2S]^2+^ mutant FDX2 and wild type FDX2 are in white. The [2Fe-2S] cluster is displayed as yellow (sulfur) and red (iron) spheres, and sidechains of prolines are displayed as white sticks. **D** On the left, backbone weighted average chemical shift differences (Δδ_avg_(HN) for residues 68-183) between [2Fe-2S]^2+^ wild type FDX2 and [2Fe-2S]^2+^ FDX2^66-183^ are reported. A chemical shift threshold value of 0.025 ppm, indicated as a dashed line, was estimated to define the significant chemical shift differences. The white bars indicate proline or unassigned NHs. Purple bars indicate the residues with chemical shift changes larger than the 0.025 threshold value. On the right, the chemical shift changes, which are larger than the 0.025 threshold value, are mapped in purple on the backbone of the crystal structure of [2Fe-2S] wild type FDX2 (residues 66-171). The backbone of prolines or unassigned residues and of the three N-terminal residues (residues 65-67) are in white. The [2Fe-2S] cluster is displayed as yellow (sulfur) and red (iron) spheres and sidechains of prolines are displayed as white sticks.

The UV/visible, UV/visible-CD and 1D ^1^H paramagnetic NMR spectra of the three recombinant proteins (wild type and mutant FDX2, and FDX2^66-183^) with their [2Fe-2S] clusters in the oxidized and/or reduced states show very similar spectral features indicative of the presence of a [2Fe-2S] cluster bound by Cys ligands (Fig. 3A and B, Supplementary Fig. 2) [22]. Overall, this comparative spectroscopic analysis indicates that the alteration of the sequence of the N-terminal stretch, as it occurs in the mutant FDX2 vs. the wild type FDX2, as well as its complete removal, as it occurs in FDX2^66-183^, do not significantly impact the cluster binding and redox properties of FDX2.

Then, we investigated whether the alteration of the N-terminal segment from the [2Fe-2S]^2+^ mutant FDX2 to the [2Fe-2S]^2+^ wild type FDX2 affects the protein structure and the backbone dynamics applying heteronuclear solution NMR spectroscopy (see Materials and Methods section for details). The NMR data showed that the N-terminal segments of both proteins adopt a random coil and highly flexible conformation (Supplementary Fig. 3) with no secondary structural elements, and that the secondary structure elements in the globular domain of the protein are the same for both proteins, reproducing those present in the crystal structure of [2Fe-2S] FDX2 (including residues Asp66-Arg171 [12]). Overall, these NMR data show that the N-terminal segments of both wild type and mutant FDX2 proteins behave as intrinsically disordered regions, without altering the folding of FDX2. These findings support the conclusion that the protein’s function is not perturbed by the different N-terminal segment present in the mutant FDX2 compared to the wild type protein.

To investigate whether the flexible N-terminal segments of wild type and mutant FDX2 transiently interact with the globular domain of FDX2 in a manner that could differentially affect their function, we compared the backbone chemical shifts of [2Fe-2S]^2+^ mutant and wild type FDX2 with those of [2Fe-2S]^2+^ FDX2^66-183^, which completely lacks this N-terminal segment. The residues in the globular domain whose chemical shifts are significantly perturbed by the presence of the N-terminal segment in the [2Fe-2S]²⁺ mutant FDX2 are primarily located in the first two β-strands adjacent to the N-terminus, as well as in the proximal loop and helix (Fig. 3C). Compared to the [2Fe-2S]²⁺ mutant FDX2, the [2Fe-2S]²⁺ wild type FDX2 shows a similar pattern of residue involvement in the globular domain due to interaction with the N-terminal segment, though the extent of this interaction is reduced (Fig. 3D). These findings indicate that the transient interaction mapped between the N-terminal segment and the globular domain in FDX2 is less pronounced in the [2Fe-2S]²⁺ wild type FDX2 than in the [2Fe-2S]²⁺ mutant FDX2. This subtle difference in the behavior of the N-terminal segment involves, however, a region of the protein located on the opposite side from the site where FDX2 interacts with its partner proteins to transfer electrons [10, 21], and thus it is expected to not significantly affect FDX2 electron transfer protein function.

In conclusion, since structural and backbone dynamic analyses indicate that the core of the mutant FDX2 found in MEOAL patient’s cells is identical to the wild type protein, it seems reasonable to assume that the functionality is also preserved.

### Biochemical and functional studies of MEOAL patient’s cells

**Fig. 4:**
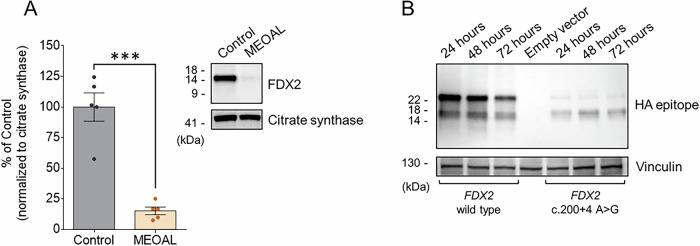
FDX2 protein levels are reduced in MEOAL patient’s cells. **A** Western blotting analysis of FDX2 in mitochondrial enriched fractions from healthy control and MEOAL fibroblasts. Equal amounts of protein lysate (i.e., 40 μg) were loaded in each lane. Protein levels were quantified after normalization with citrate synthase, used as loading control, and expressed as a percentage of healthy control. Reported data result from the mean of five independent experiments ±SEM and statistical significance was determined using unpaired t-test (***p ≤ 0.001, compared to control). **B** Comparative assessment of exogenous expression levels of wild type and mutant FDX2 in transfected HEK293T cells, analyzed by western blot. A representative image of one of three independent experiments is reported. For the detection of the proteins, an anti-HA epitope primary antibody was used. Protein expression was monitored at 24, 48, and 72 h post-transfection, loading the same amount of protein lysate (i.e., 60 μg) in each lane and using vinculin as loading control.

We next determined the level of FDX2 in mitochondrial fractions from cultured fibroblasts of the MEOAL patient by western blotting and found low amounts compared to fibroblasts from the healthy control (Fig. 4A). Consistent with the only two residues longer mutant FDX2, its molecular weight was similar to the wild type protein. To further address the effects of the c.200+4 A > G mutation on the expression of the FDX2 protein, HEK293 cells were transiently transfected with a plasmid containing either the sequence of the wild type *FDX2* or the sequence with the c.200+4 A > G mutation, both fused at the C end with a HA epitope coding sequence to allow western blotting analyses of the recombinant proteins, as described in Materials and Methods. We found very low levels of mutant FDX2, both in the unprocessed and in the mature forms, compared to the wild type protein (Fig. 4B), in the entire time course up to 72 h.

#### The c.200+4 A > G mutation in FDX2 affects the biogenesis of FeS clusters and mitochondrial respiration in MEOAL patient’s cells

**Fig. 5:**
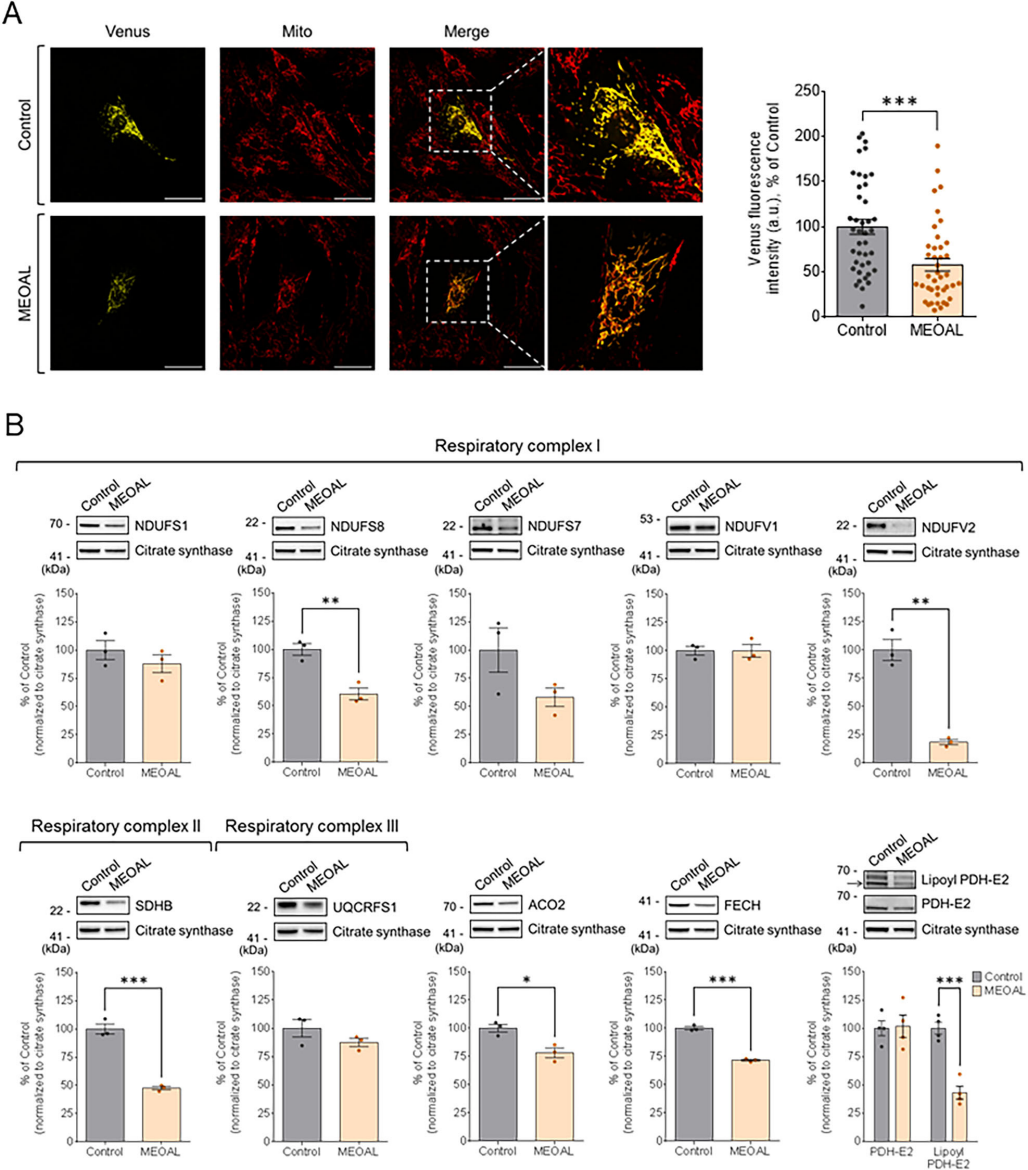
Biosynthesis of FeS clusters is impaired in MEOAL cells. **A** On the left, representative confocal images of healthy control and MEOAL fibroblasts co-transfected with the complementation system expressing the two Venus fragments fused to GRX2. Venus fluorescent signal (yellow) co-localizes within mitochondria, as confirmed by its overlap with the fluorescent signal derived from immunofluorescence staining of respiratory complex I (red). Scale bar: 60 μm. On the right, quantitative analysis of Venus fluorescent signal intensity expressed as a percentage of healthy control. Data are reported as the mean of three independent experiments ±SEM and statistical significance was determined using unpaired t-test (***p ≤ 0.001, compared to control). **B** Western blotting analysis of mitochondrial proteins in whole cells extracts from healthy control and MEOAL patient’s fibroblasts. Equal amounts of protein (i.e., 40 μg) were loaded in each lane. Protein levels were quantified after normalization with citrate synthase and expressed as a percentage of control levels. Reported data result from the mean of at least three independent experiments ±SEM and statistical significance was determined using unpaired t-test (*p ≤ 0.05, **p ≤ 0.01, ***p ≤ 0.001 compared to healthy control).

As reported in the Introduction, FDX2 has a key role in the biosynthesis of the FeS clusters, and we therefore addressed the effects of the c.200+4 A > G mutation both in this pathway and in the steady-state levels of several mitochondrial proteins containing these prosthetic groups. At first, we analyzed the formation of [2Fe-2S] clusters in control and MEOAL cells by taking advantage of a fluorescence imaging tool, kindly provided by Jonathan Silberg (Rice University, Houston, USA). This system is based on two fusion proteins, one carrying the N-terminal half of the Venus yellow fluorescent protein fused to a FLAG-tagged human glutaredoxin 2 (GRX2) protein and another carrying the Venus C-terminal half fused to GRX2 as well. A presequence for the import into the mitochondria, where a large fraction of FeS clusters is located, is present in both constructs. When expressed in the same cell, the chimeric protein exhibits fluorescence only after GRX2 homodimerization, a process quantitatively dependent upon the insertion of a bridging [2Fe-2S] cluster [23]. Control and MEOAL cells were transiently co-transfected with the constructs allowing the expression of the two Venus/GRX2 fusion proteins, as described in Materials and Methods. Western blotting analysis using an anti-FLAG antibody indicates similar transfection efficiency in control and MEOAL cells (Supplementary Fig. 4A).Confocal images reveal that Venus’s fluorescence localizes within mitochondria, both in control and in MEOAL patient’s cells (Fig. 5A). Experiments carried out by transfecting cells with the individual constructs resulted in the complete absence of Venus signal (Supplementary Fig. 4B). Visual inspection of several images followed by the quantitative analyses described in detail in Materials and Methods shows that the Venus fluorescence is significantly decreased in patient’s cells, in line with an impaired [2Fe-2S] cluster biosynthesis due to the mutation of FDX2.

The relative abundance of various mitochondrial FeS proteins was examined in control and MEOAL patient’s cells by quantitation of western blots. The apoforms of FeS proteins are frequently instable and as a result they become degraded [12, 24]. The FeS subunits of respiratory complexes I and II (NDUFS8, NDUFV2, SDHB) as well as aconitase (ACO2) and ferrochelatase (FECH) were decreased (relative to citrate synthase as mitochondrial control protein) in MEOAL cells (Fig. 5B). Likewise, we noted a defect in lipoylation, presumably due to defective maturation of the FeS protein lipoyl synthase (LIAS), as evident from immunostaining of the lipoyl cofactor of the E2 subunit of PDH (Fig. 5B). Several other mitochondrial proteins were either unchanged or decreased only weakly (Supplementary Fig. 5). Collectively, the data indicated a significant decrease in the amounts of several mitochondrial FeS proteins suggesting impaired maturation in MEOAL cells as a likely reason.

**Fig. 6:**
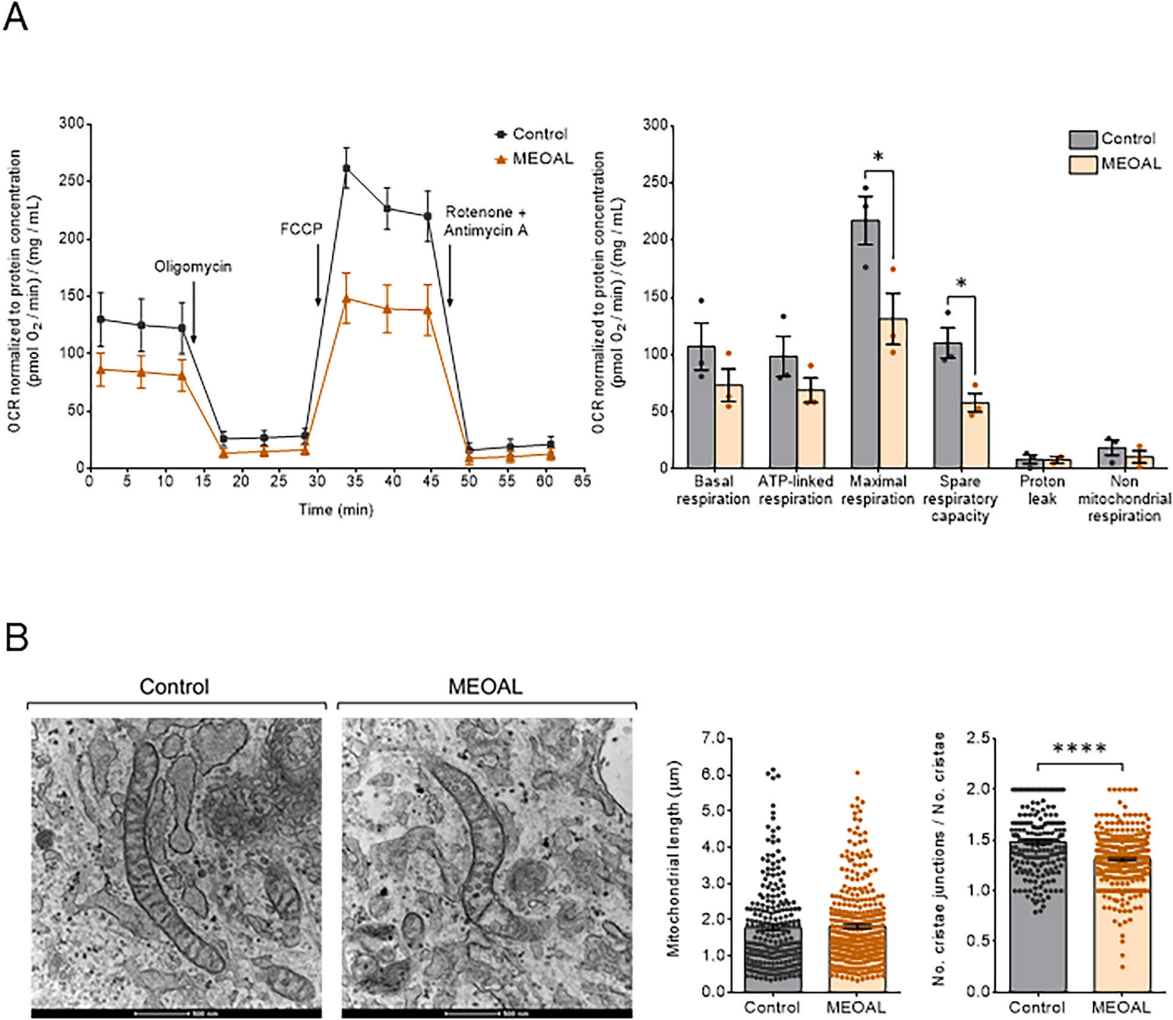
Bioenergetics and mitochondrial ultrastructure are altered in MEOAL cells. **A** Oxygen consumption rates (OCR) of fibroblasts were measured in real time under basal conditions and after injection of oligomycin, FCCP, and rotenone + antimycin A, as indicated in the figure. Values were normalized to the total protein concentration (mg/mL). Bioenergetic parameters, on the right side of the panel, were calculated as described in Material and Methods. Reported data result from the mean of three independent experiments ±SEM and statistical significance was determined using unpaired t-test (*p ≤ 0.05). **B** On the left, representative electron micrographs of healthy control and MEOAL mitochondria. Cells were fixed and TEM images of randomly selected fields were acquired. Scale bars: 0.5 μm. On the right, morphometric analysis of mitochondrial length and ratio between number of cristae junctions and number of cristae per mitochondrion. Data, reported as the mean value ± SEM, derived from four independent experiments in which a total of 233 mitochondria for the control cell line and 339 mitochondria for the patient cell line were analyzed. Statistical significance was determined using unpaired t-test (****p ≤ 0.0001, compared to control).

We next measured the oxygen consumption rate of MEOAL patient’s cells by means of a Seahorse flux analyzer and found, in comparison to control, a decrease in maximal respiration and spare respiratory capacity, with no significant difference both in basal and in ATP-linked respiration (Fig. 6A). These data indicate an impaired capability of MEOAL cells carrying the c.200+4 A > G mutation in *FDX2* to boost the mitochondrial oxygen consumption rate in conditions of increased energy demand, consistent with the decreased levels of multiple respiratory subunits reported above. We further analyzed the enzymatic activities of the respiratory complexes in MEOAL patient’s cells, either individually (I, II, III, and IV) or in combination (II + III), by spectrophotometric assays. There was a trend for lower activities in MEOAL cells, although not statistically significant (Supplementary Fig. 6). Nevertheless, the data on Venus/GRX2 maturation, FeS proteins levels, and respiratory enzyme activities indicate a clear, even though weak FeS proteins biogenesis defect in MEOAL cells.

Mitochondrial functions are intimately linked with their ultrastructure and in particular with the dynamic shape of the cristae, a subcompartment of the inner membrane essential for energy transduction [25]. Defects in FeS proteins biogenesis are frequently associated with severe alterations in mitochondrial ultrastructure [15, 26], and we therefore compared mitochondria from healthy control with MEOAL patient’s cells by means of transmission electron microscopy. Figure 6B indicates that mitochondria from patient’s cells possess a higher number of fragmented cristae as indicated by a decreased ratio between cristae junctions and cristae number. The overall length of mutant mitochondria was comparable to control cells. Collectively, also these ultrastructural data are consistent with an impaired FeS proteins biogenesis combined with a lower respiratory activity in patient’s cells.

#### MEOAL patient’s cells with c.200+4 A > G mutation in *FDX2* show enhanced mitochondrial iron accumulation and ROS production

**Fig. 7:**
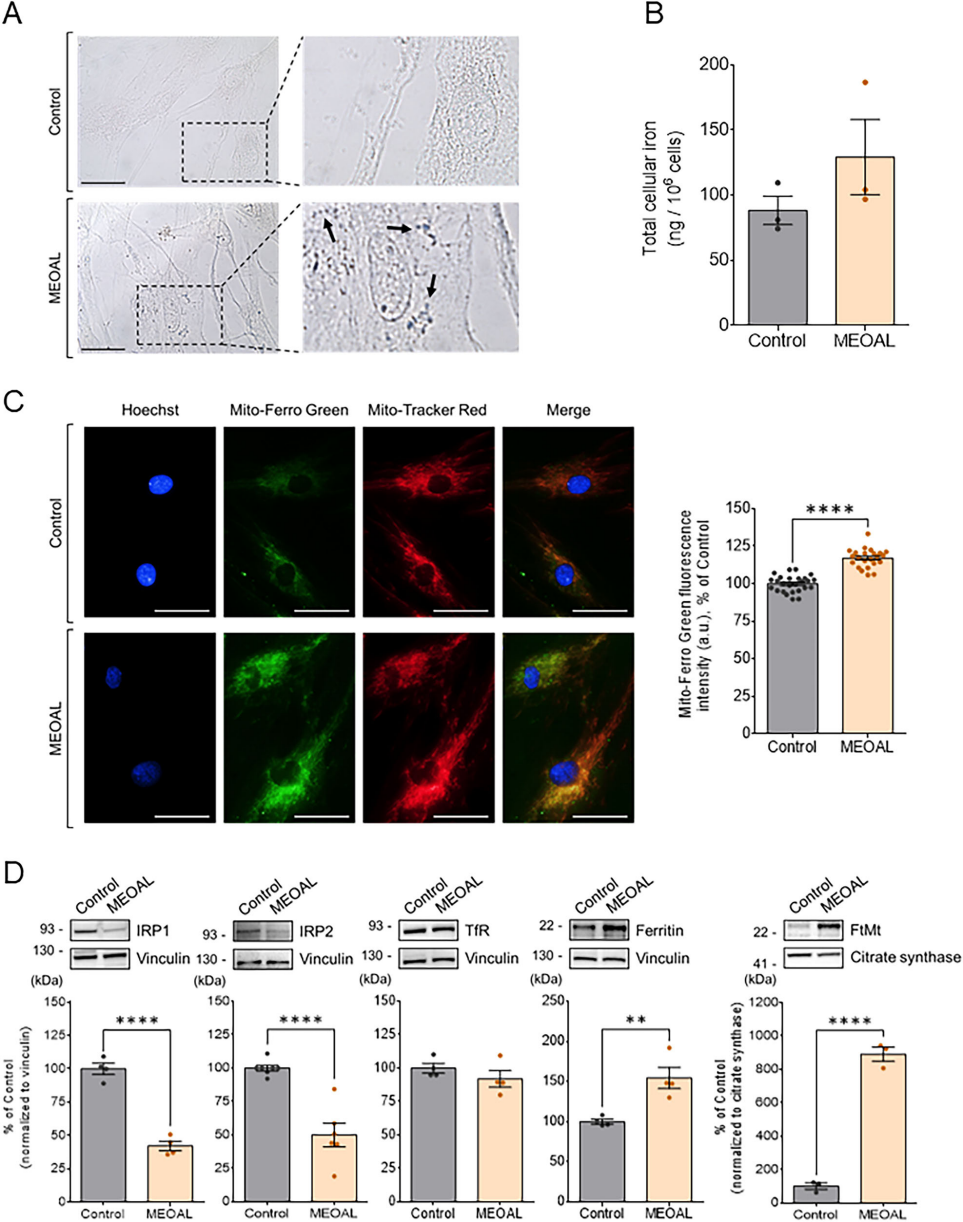
Iron homeostasis is dysregulated in MEOAL cells. **A** Prussian blue staining reveals iron deposits as blue dots in MEOAL fibroblasts in comparison to healthy cells (for sake of clarity, blue deposits are indicated by black arrows in the field magnification). Scale bar: 30 μm. **B** Quantitative analysis by ICP-MS of total intracellular iron in healthy and MEOAL fibroblasts. Reported data result from the mean of three independent experiments ±SEM. Statistical significance was determined using unpaired t-test (non statistically significant, compared to control). **C** Relative levels of mitochondrial labile iron were analyzed in healthy and MEOAL cells by means of the fluorescent probe Mito-FerroGreen. On the left, representative fields confirming the colocalization of Mito-FerroGreen fluorescence signal (green) with Mito-Tracker Red (red). Nuclei were counterstained with Hoechst (blue). Scale bar: 50 μm. On the right, quantitative analysis of Mito-FerroGreen fluorescence signal intensity expressed as a percentage of healthy control. Data are reported as the mean ± SEM of three independent experiments and statistical significance was determined using unpaired t-test (****p ≤ 0.0001, compared to control). **D** Western blotting analysis of key proteins involved in iron homeostasis. Equal amounts of protein lysate (i.e., 40 μg) were loaded in each lane. Protein levels were quantified after normalization with vinculin, used as loading control, and expressed as a percentage of healthy control. In each graph the mean value ± SEM of at least three independent experiments is reported; statistical significance was determined using unpaired t-test (**p ≤ 0.01, ****p ≤ 0.0001 compared to healthy control).

Iron overload has been detected in several diseases associated with mutations in proteins involved in the FeS proteins biogenesis [27], such as Friedreich ataxia [28–30], ISCU2 myopathy [31], GLRX5-related disease [32] and, more recently, in a neuromuscular disorder caused by a loss-of-function mutation in CIAO1, a key component of the cytosolic FeS clusters assembly complex [33]. In cultured cells, RNAi-mediated silencing of mitochondrial genes involved in the de novo [2Fe-2S]-cluster assembly (core ISC genes), in particular of *FDX2*, has been shown to lead to increased mitochondrial iron accumulation [15]. To address this issue in MEOAL disease, we first exploited Prussian blue staining to detect ferric iron and found in patient’s cells a distinctive punctate pattern suggesting iron deposits (indicated by the arrows in Fig. 7A), which are completely undetectable in control cells. Measurement of the total cellular iron accumulation by ICP-MS (Inductively Coupled Plasma Mass Spectrometry) showed a moderate increase in the iron content of the MEOAL culture sample compared to control (Fig. 7B). Possibly, the iron deposition occurred only in a subset of the cultured MEOAL cells. Mitochondria, being the major site of heme and FeS cluster biosynthesis [34–37], are also the main cellular iron users and play a central role in cellular iron metabolism via ISC-dependent regulatory mechanisms. To evaluate the iron loading of these organelles in MEOAL cells, we treated control and patient’s cells with Mito-FerroGreen, a fluorescent mitochondria-targeted probe that labels “free” ferrous ion belonging to the labile iron pool (i.e., the pool of redox-active chelatable iron, comprising both Fe^2+^ and Fe^3+^ ionic forms) [38]. Co-localization of Mito-FerroGreen signal with MitoTracker Red label confirms the mitochondrial targeting of the probe (Fig. 7C). Quantitative analyses of confocal images, performed as described in Materials and Methods, indicated a small, yet significant iron accumulation in mitochondria of MEOAL patient cells compared to control, similarly to what has been shown for FDX2-depleted cultured cells [15].

In healthy cells, iron overload can be counteracted by transport and storage systems that keep the concentration of “free” iron at physiological needs. Cellular iron levels are post-transcriptionally regulated by iron regulatory proteins 1 (IRP1, also known as ACO1) and 2 (IRP2, also known as IREB2). IRP1 and IRP2 sense cellular iron levels indirectly via FeS cluster-dependent pathways that modulate the expression of proteins involved in iron uptake, storage and delivery, including transferrin receptor 1 (TfR1) and ferritin, by interacting with *cis*-regulatory iron responsive elements (IREs) in the 5’ or 3’ untranslated region of the target RNAs [37, 39, 40]. IRP1 is mainly modulated by reversible FeS cluster binding, whereas IRP2 is regulated by iron-dependent proteasomal degradation initiated by FBXL5 which also binds FeS clusters [41, 42]. In healthy cells, the joint action of IRP1 and IRP2 allows a fine modulation of cytosolic iron levels: in conditions of iron deficiency, levels of apo-IRP1 and of IRP2 protein increase. Their binding to IREs leads to TfR1 transcript stabilization and ferritin translational repression, and results in an increase of iron uptake and decrease of its storage; in iron-replete states, IRP1 contains a [4Fe-4S] cluster and loses the capability to interact with IREs, and IRP2 becomes a target of FBXL5 for proteasomal degradation. This lowers iron uptake by the cell, due to the decrease of TfR1, and promotes iron storage into ferritin.

To explore the status of iron regulation in MEOAL patient’s cells, we analyzed the levels of IRP1 and IRP2 as well as of their targets TfR1 and ferritin. Western blotting analyses indicated that IRP1 and surprisingly also IRP2 were decreased in patient’s cells compared to control (Fig. 7D). For IRP1 this result is consistent with an increase of the degradation-sensitive apoform [43, 44]. For IRP2, core ISC deficiency usually results in elevated levels, whereas defects in CIA lead to lower levels [24]. The reason for the unexpected result for IRP2 in MEOAL cells remains unclear. Also, the comparative analysis of the levels of TfR1 and ferritin provided unexpected results: while the levels of TfR1 were unchanged in patient’s cells relative to control, we found higher rather than the expected lower levels of ferritin in patient’s cells compared to control (Fig. 7D). Further studies are needed to unravel the aberrant regulatory mechanisms in these cells.

We additionally analyzed the expression levels of the mitochondrial ferritin (FtMt), since it has been proposed to work in several tissues, including brain, as an iron storage protein in a similar way to its cytosolic counterpart to capture excess labile iron in a redox-inactive form [45, 46]. Of note, FtMt expression does not rely on the IRPs’ modulation. Figure 7D shows that the levels of FtMt were drastically increased in MEOAL patient’s cells compared to control. In combination with the MitoFerroGreen fluorescence assay (Fig. 5A) and the mitochondrial FeS proteins defects, this result suggests that excess iron, which cannot be used to produce FeS clusters due to the deficiency of FDX2, may only partially be bound to FtMt, thus potentially contributing to the pool of toxic “free” redox-active chelatable iron in mitochondria.

**Fig. 8:**
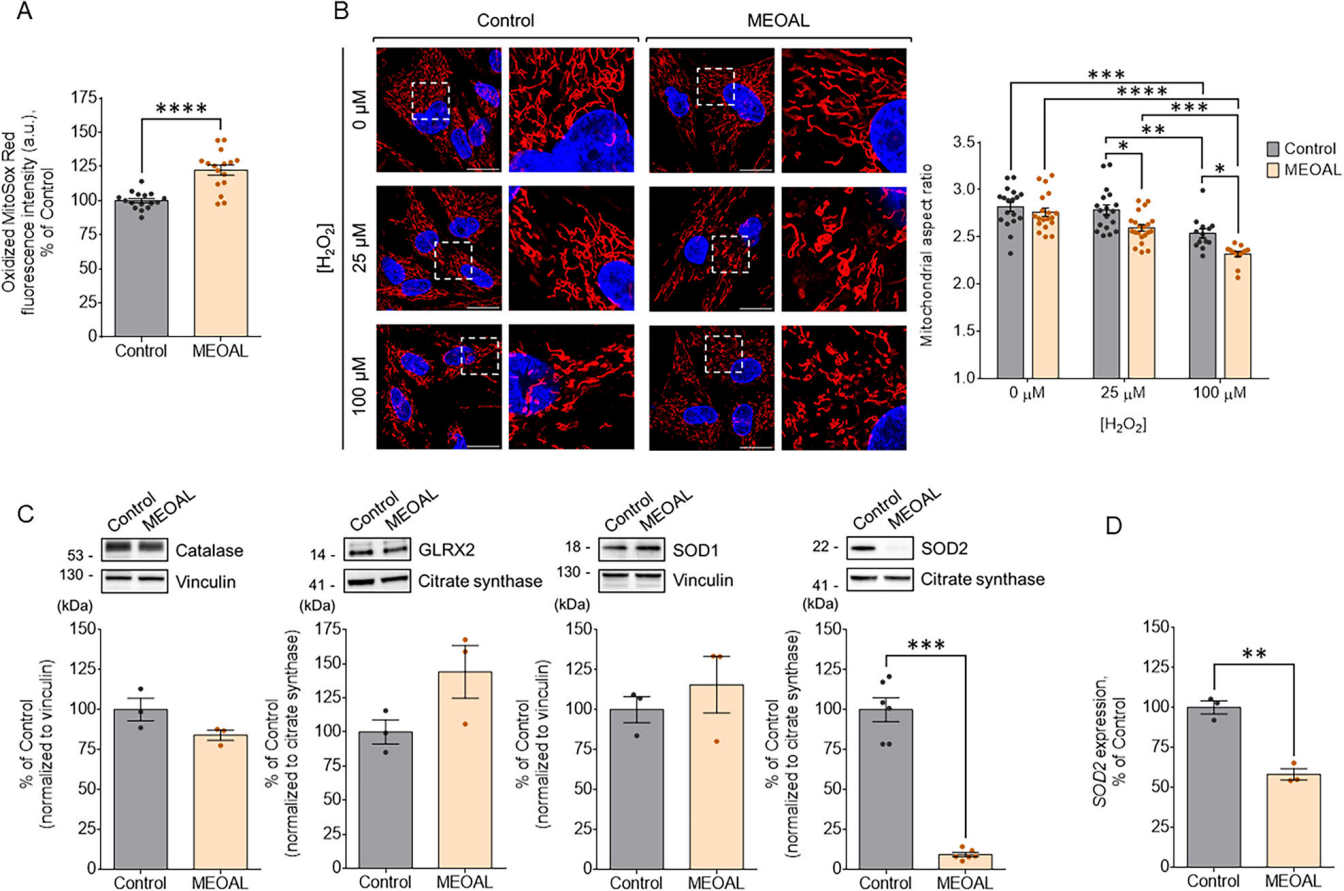
MEOAL cells exhibit increased susceptibility to oxidative stress. **A** Mitochondrial ROS levels were evaluated by incubating healthy and MEOAL fibroblasts with MitoSox Red and analyzing the fluorescent signal upon the oxidation of the probe. Data are reported as the mean ± SEM of three independent experiments and expressed as a percentage of healthy control. Statistical significance was determined using unpaired t-test (****p ≤ 0.0001, compared to control). **B** Susceptibility to oxidative stress of healthy and MEOAL cells was assessed by analyzing the mitochondrial fragmentation upon treatment of cells with two concentrations of H_2_O_2_ for 2 h. On the left, representative confocal images of healthy control and MEOAL fibroblasts treated with or without H_2_O_2_ in which mitochondria were stained with a primary antibody against respiratory complex I (red) and nuclei were counterstained with DAPI (blue). On the right, quantitative analysis of mitochondrial aspect ratio. Reported data are expressed as mean of four independent experiments ±SEM and statistical significance determined using Two-way ANOVA with Tukey’s multiple comparisons test (*p ≤ 0.1, **p ≤ 0.01, ***p ≤ 0.001 and ****p ≤ 0.0001). **C** Western blotting analysis of key proteins involved in antioxidant defense. The same amount of protein lysate (i.e., 40 μg) was loaded in each lane. Protein levels were quantified after normalization with vinculin and expressed as a percentage of healthy control. Data represents the mean value of at least three independent experiments ± SEM and statistical significance was determined using unpaired t-test (***p ≤ 0.001, compared to healthy control. **D** SOD2 expression measured by qRT-PCR in heathy control and MEOAL patient’s cells. SOD2 expression levels are normalized to those detected in control cells, expressed as 100%. Analyzed data are reported as the mean of three independent experiments ± SEM and statistical significance determined using unpaired t-test (**p ≤ 0.01, compared to healthy control).

Iron overload can potentially trigger the generation of reactive oxygen species (ROS) [47]. We therefore investigated whether the abnormal iron handling in mitochondria of MEOAL cells may result in increased generation of ROS in this compartment. To this end, control and patient’s cells were incubated with MitoSOX Red, a probe specifically targeted to mitochondria and exhibiting red fluorescence upon oxidation by superoxide. Comparative analyses showed that the fluorescence signal of the oxidized MitoSOX Red sensor was significantly higher in MEOAL patient’s cells compared to control, suggesting a mildly increased mitochondrial basal production of ROS (Fig. 8A). Consistently, when subjected to increasing concentrations of H_2_O_2_ as pro-oxidant stimulus, patient’s cells showed slightly more fragmented mitochondria compared to control, as assessed by a decreased aspect ratio (i.e., mitochondrial length/width) evaluated by confocal microscopy (Fig. 8B).

We finally examined by western blot the expression level of several proteins involved in the cellular antioxidant defense, i.e., catalase, glutaredoxin 2 (GLRX2), superoxide dismutase 1 (SOD1) and mitochondrial superoxide dismutase 2 (SOD2). The levels of catalase, GLRX2 and SOD1 were not significantly increased in MEOAL cells compared to control, showing that the stress response to the FDX2 mutation was rather mild (Fig. 8C). This may fit into the weak FeS proteins biogenesis defect and the small mitochondrial iron overload in MEOAL cells, producing mild oxidative stress. Interestingly, the levels of SOD2 were tenfold diminished (Fig. 8C), i.e., much more than expected from the 40% diminution of SOD2 mRNA levels in MEOAL cells as estimated by qRT-PCR (Fig. 8D), indicative for a substantial protein destabilization. The rather low matrix content of active SOD2 may partially explain the increased ROS signals detected above by MitoSOX Red approach.

## Discussion

Ferredoxin 2 (FDX2) is a mitochondrial protein with a crucial role in providing reducing equivalents for the biosynthesis of FeS clusters [7–10]. The latter are ubiquitous metallo-cofactors performing essential and different biochemical functions (including redox catalysis, β-oxidation of lipids, gene expression regulation, and DNA repair/replication) [48]. It is therefore not surprising that a number of human diseases are associated with defects in their biosynthesis (see [49] for a comprehensive review in this topic). Among these diseases, MEOAL is an inherited neuromuscular disorder caused by homozygous mutations of *FDX2*. To date only 11 patients have been reported presenting clinical phenotypes with variable onset and severity, ranging from muscle weakness and exercise intolerance with episodic exacerbation to a more complex neurological involvement which may include optic atrophy, reversible or partially reversible leukoencephalopathy and later onset of a sensory-motor polyneuropathy [1–6]. Table S1 (Supplementary material) provides a summary of the molecular, clinical, and neurological findings from the patients reported to date by the medical literature and includes the one described in this work. Although MEOAL has been unequivocally associated with FDX2 mutations, to date a clear cause-effect relationship has not been established, the specific contribution of this protein to the pathology onset and progression has not been clarified and no therapeutic measures are known to modify the natural history of the disease.

In this work we describe the effects of a mutation in *FDX2*, i.e., c.200+4 A > G, that we identified in a pediatric patient with a severe neurological condition showing ataxic features, hyposthenia, and pyramidal signs. We found that this mutation results in an altered protein, which is present at low levels in patient’s cells (Fig. 4A).

By means of spectroscopic analysis on recombinant proteins purified from *E. coli*, we evaluated the structural properties of the mutant FDX2 protein and, more in general, the effect of the alteration of the N-terminal portion of the protein compared to wild type. To this end, we compared the wild type FDX2 with two different constructs with an altered N-terminal moiety: (i) the mutant FDX2 and (ii) a protein in which the N-terminus was completely removed (i.e., FDX2^66-183^) (Supplementary Fig. 1). Overall, the structural and dynamic properties of the N-terminal segment of the [2Fe-2S] mutant FDX2 closely resemble those of the wild type protein, and even the removal of this segment does not perturb them (Fig. 3C and D and Supplementary Fig. 3). The cluster binding and redox properties are not affected by the different N-terminal segments present in the mature wild type and mutant FDX2 or by its absence, in line with the significant structural distance of the N-terminal segment from the cluster ligands (Fig. 3A and B and Supplementary Fig. 2). These findings suggest that the sequence modification at the N-terminus introduced by the mutation, or by its deletion, does not dramatically alter the function of FDX2. Of note, this is consistent with the previous finding that the expression of FDX2^66-183^ is able to almost completely rescue the growth of a mutant yeast strain deleted of Yah1, the homolog of human FDX2 [15]. Therefore, our data confirm that the N-terminal region of mature FDX2 is not essential for its function in the electron transfer chain required for the biosynthesis of FeS clusters.

Taken together, these results suggest that any potential alteration found in MEOAL patient cells carrying the c.200+4 A > G mutation in *FDX2* is likely due to the decrease of the FDX2 protein levels rather than to a structural alteration of the mature protein. The two other pathogenic mutations of *FDX2* known to date to be associated with MEOAL (i.e., c.1 A > T and c.431 C > T) also lead to severe diminution of FDX2 and therefore this patient cell line provides an appropriate model to investigate the molecular mechanisms underlying the disease pathophysiology. We found that cells from the FDX2-deficient patient described in this work have an affected mitochondrial phenotype. We observed a decrease in mitochondrial FeS proteins content in patient’s cells compared to age-matched healthy controls (Fig. 5B), consistent with diminished levels of FDX2. Although single FeS protein activities were only weakly diminished, the combined respiratory chain defect was significantly impaired (Fig. 6A).

Disruption of cellular iron homeostasis has been proposed to contribute to the pathogenesis of several neurodegenerative diseases, including those associated with dysfunctions in the FeS clusters assembly [27–32]. Consistently, we found an increase of total iron (Fig. 7A and B) and a significant iron accumulation in mitochondria of MEOAL patient cells compared to healthy controls (Fig. 7C). In physiological conditions, iron homeostasis and crosstalk between cytosol and mitochondria are finely tuned by the coordinated action of iron regulatory proteins IRP1 and IRP2, which sense iron accumulation in the cytosol and ensure that cells acquire adequate amounts for their needs without reaching toxic levels [37, 39, 40]. The western blotting analysis of IRPs and of their targets ferritin and TfR1 in patient and healthy cells provided intriguing results. Both IRPs are decreased in patient cells compared to control, a result that fits to the current model of ISC-dependent iron regulation for IRP1, but not IRP2 (Fig. 7D). Also the unchanged levels of TfR1 and the higher rather than the expected lower levels of ferritin in patient’s cells relative to control do not fit to the classical model of cellular iron regulation [24], requiring further studies on these unusual findings.

Interestingly, we found a significant increase in the levels of the mitochondrial ferritin (FtMt) in patient’s cells compared to control (Fig. 7D). FtMt has a comparable iron-storage role as cytosolic ferritin, but its expression in healthy tissues is low and restricted to those with high energy demand, including brain [46], and does not rely on regulation by IRPs. FtMt has attracted increasing attention, as its misregulation has been observed in several neurological disorders, such as Alzheimer and Parkinson diseases, restless legs syndrome, Friedreich ataxia and age-related macular degeneration [50–54]. Of note for our work, it has been previously shown that overexpression of FtMt can impact cellular iron homeostasis by driving iron delocalization from cytosol to mitochondria [50, 55, 56]. The presence of “free” chelatable iron in mitochondria of MEOAL patient cells (Fig. 7C) suggests that the storage capacity of FtMt is overcome and this could explain both the increased mitochondrial basal production of ROS that we observed by MitoSoxRed staining (Fig. 8A) and the higher sensitivity to pro-oxidant conditions (Fig. 8B), as previously found in other neurodegenerative diseases associated with dysfunctional FeS clusters assembly process, such as Friedreich ataxia [57]. The increased expression of FtMt in several neurological disorders has been proposed to relate to a potential neuroprotective role against iron overload and oxidative stress, whereas in others, such as the restless legs syndrome, it may link to the onset of the disease rather than to neuroprotection [53]. Although further studies are needed to clarify the relationship between FtMt expression and the abnormal iron metabolism/oxidative stress in MEOAL patient’s cells, our data suggest that the increase of FtMt may be a response to the pathogenesis by low FDX2 levels.

In physiological conditions, ROS production is counterbalanced by antioxidant systems to maintain the redox homeostasis in the cell. We therefore analyzed the expression of selected antioxidant proteins in MEOAL patient’s cells and in control cells. Interestingly, while the levels of catalase, glutaredoxin 2 and superoxide dismutase 1 (SOD1) are unaffected, those of superoxide dismutase 2 (SOD2) are significantly decreased in patient’s cells (Fig. 8C). While SOD1 is localized in the cytosol and in the mitochondrial intermembrane space, SOD2 is exclusively present in the mitochondrial matrix [58], and this is particularly relevant considering the results shown in this work: SOD2 is a first-defense mitochondrial antioxidant enzyme, and its decrease could be a specific alteration impairing the response of MEOAL patient’s cells to pro-oxidant stimuli. Mitochondrial antioxidant systems are critically involved in neurodegenerative disorders in which ROS toxicity is a major pathogenic factor, and SOD2 has been claimed to participate in the progression of neurodegenerative disorders such as stroke and Alzheimer and Parkinson diseases [59–61]. It is worth noting that a decrease of SOD2 levels has been found in various cellular and animal models of Friedreich ataxia [62–64], which shares several biochemical features with MEOAL including iron overload and increased sensitivity to oxidative stress likely due to defects in FeS clusters biosynthesis [57, 65, 66]. Strikingly, in most cases Friedreich ataxia cells show a significant decrease in SOD2 mRNA as well, which we also found in the MEOAL patient’s cells characterized in the present work (Fig. 8D). This might suggest a common behavior of SOD2 in neurodegenerative diseases associated with FeS clusters deficiency, leading to a lower tolerance to oxidative stress. It is also worth noting that independent studies have previously shown that iron overload inactivates SOD2 in yeast, and a similar effect was reported in a mouse model of hereditary hemochromatosis [67–69]. SOD2 specifically binds manganese as metal cofactor to carry out its catalytic activity, but when mitochondrial iron homeostasis is disrupted, as in cells with defects in FeS cluster assembly, iron accumulated in a reactive form could compete with manganese for binding to SOD2, inactivating the enzyme [69]. Experiments addressing the potential loss of SOD2 activity due to misincorporation of iron are currently under way in our laboratory. Understanding the SOD2 downregulation and potential iron-mediated mis-metalation of the corresponding protein could open a window for further studies aimed at investigating SOD2 as a potential transcriptional and post-transcriptional target underpinning the MEOAL pathophysiology, and in turn help to boost therapeutic progress, which is an urgent unmet need.

## Materials and methods

All methods were performed in accordance with the relevant guidelines and regulations.

### Neuroimaging

Brain MRIs were performed with an MR 1.5 (4 and 5 years old) or 3 T scanner (Signa Premiere 3 T GE Healthcare) when the girl was 6 and 8 years old. The whole protocol includes: axial Spin Echo T1-weighted images (repetition time 540 ms; Echo time 10 ms; slice thickness 4 mm; field of view 24 × 29.6 cm; acquisition matrix 192\256, Number NEX 2, flip angle: 74); Fast spin Echo T2 (repetition time 5777 ms; Echo time 100,23 ms; slice thickness 4 mm; spacing 5 mm; field of view 24 × 29.6 cm; acquisition matrix 512\512, NEX1, flip angle: 111); Coronal fast spin echo T2 (repetition time 7884 ms; Echo time 101.55 ms; slice thickness 3.50 mm; spacing 2 mm; field of view 24 × 29.6 cm; acquisition matrix 416\512, NEX1, flip angle: 111); 3D-Flair (repetition time 8002 ms; Echo time 128.47 ms; slice thickness 1 mm; spacing 1 mm; field of view 24 × 29.6 cm; acquisition matrix 240\240, NEX1, flip angle: 90); 3D-MPRAGE T1: (repetition time 2471.74 ms; Echo time 3.82 ms; slice thickness 1 mm; spacing 1 mm; field of view 22 × 27.1 cm; acquisition matrix 220\220, NEX2, flip angle: (8); SWAN (repetition time 39.70 ms; Echo time 24.28 ms; slice thickness 1.95 mm; spacing 1 mm; field of view 22 × 27.1 cm; acquisition matrix 300\300, NEX0,70, flip angle: 15); Axial DTI b1000 30DIR (repetition time 3100 ms; Echo time 59.90 ms; slice thickness 4 mm; spacing 5 mm; field of view 24 × 29.6 cm; acquisition matrix 128\192, NEX 1, flip angle: 90).

### Cell lines and culture conditions

Patient’s primary fibroblasts were obtained from a skin biopsy. The “Cell line and DNA bank of Genetic Movement Disorder and Mitochondrial Diseases” (GMD-MDbank), member of the Telethon Network of Genetic Biobanks (project no. GTB12001), funded by Telethon Italy, and EuroBioBank network, provided us with specimens. Control primary fibroblasts from a healthy age-matched subject were kindly provided by Dr. Erika Fernandez-Vizarra, University of Zaragoza, Spain [70]. Isolated cells were maintained in culture with DMEM high-glucose supplemented with GlutaMAX™ (GIBCO Life Technologies), 10% (v/v) heat-inactivated FBS (GIBCO Life Technologies), 100 U/mL penicillin and 100 µg/mL streptomycin (GIBCO Life Technologies), under 5% CO_2_ at 37 °C. For cell passaging, fibroblasts were washed with Phosphate Buffered Saline (PBS), detached with 0.25% trypsin/EDTA (GIBCO Life Technologies) and seeded with fresh growth medium.

### DNA and RNA-sequencing

The NGS molecular analysis of patient’s genomic DNA was performed by targeted sequencing of bridge-PCR products captured by a panel of probes designed with KAPA HyperCap technology (Roche). The sequencing platform utilized was a NextSeq500 Illumina. The sequence alignments were performed with Burrows-Wheeler Aligner (BWA) software (version 0.7.15), while the variant calls were made by using the HaplotypeCaller tool belonging to the Genome Analysis Toolkit (GATK) suite (version 4.0). The annotation of variants was made using the QCI Interpret software (Qiagen). Interpretation of variants identified by this analysis was based on the current knowledge and on the American College of Medical Genetics and Genomics (ACMG) classification [71]. The variants reported with a frequency greater than 1% in the gnomAD database, classified as benign or probably benign according to the ACMG criteria or of uncertain significance in autosomal recessive genes were not considered. For FDX2 transcript analysis, total RNA was extracted from cultured skin fibroblasts and retrotranscribed as reported in [71]. FDX2 mRNA was amplified using primers FDX2_ex1_F 5’-CGTGAGTGCCAGGGTTCTAC-3’ and FDX2_ex5_R 5’-GTGTTCATGTCAGTGGGGCT-3’. PCR products were visualized using a 1% agarose gel. Densitometric analysis was performed using ImageJ software. Bands were excised from the gel and sequenced using amplification primers. An aliquot of the PCR reaction was also sequenced using an Illumina MiniSeq desktop sequencer and the Nextera Library Preparation Kit as reported in [72].

### Real Time PCR

SOD2 quantification was performed by Real Time PCR from 20 ng of cDNA obtained from control and patient’s fibroblasts, as reported in [72]. The analysis was performed using the ViiA 7 Real-Time PCR System (Thermo Fisher Scientific), the SensiFast SYBR No Rox kit (Meridian), and primers for *SOD2* (F: 5′-CTGATTTGGACAAGCAGCAA-3′, R: 5′-CTGGACAAACCTCAGCCCTA-3′) and GAPDH as a housekeeping gene (F: 5’-TCCTCTGACTTCAACAGCGA-3’, R: 5’-GGGTCTTACTCCTTGGAGGC-3’). The quantification of *SOD2* relative to *GAPDH* was performed following the ΔΔCt method.

### Western blotting

For immunoblot assays, cells have been harvested at similar confluence. Membranes were incubated with the primary antibodies listed in Table S2 (Supplementary material) and then with HRP-conjugated secondary antibodies. Dilution and time of incubation of each antibody are reported in Table S2. Proteins were detected using Immobilon® Forte Western HRP Substrate (Millipore) by Imager CHEMI Premium Detector (VWR). Reaction product levels were quantified with ImageJ Fiji software and normalized to those of vinculin or citrate synthase.

### HEK293T transfection

The pcDNA 3.1 (+) vectors containing the human FDX2 precursor coding sequence and the human FDX2 precursor coding sequence with c.200+4 A > G mutation were purchased at GenScript Biotech B.V. (Leiden, Netherlands). Both sequences were fused at the 3’ end to a coding sequence for HA epitope for Western blotting analysis. Transient transfections were performed using Lipofectamine^TM^ 2000 (Life Technologies) in OptiMEM (GIBCO Life Technologies), at 70% cell confluency. After 8 h, the transfection medium was removed, and cells were maintained in culture in standard growth conditions. The expression levels of recombinant proteins were assessed after 24, 48, and 72 h, collecting cells and performing a Western blotting analysis with an anti-HA epitope antibody.

### FeS cluster fluorescent assay

The content of 2Fe-2S clusters in cells was evaluated using the experimental method developed by Hoff and coworkers [23]. To this purpose, 40 × 10^3^ fibroblasts/well were seeded on 13 mm diameter cover glasses in 24-well plate to reach approximately 80% final confluence. Cells were co-transfected with the N- and C-terminal Venus-GRX2 fusion plasmids. Vector expressing N173 was designed to generate a fusion protein with C-terminal FLAG tag for Western blotting analysis. Transfection was performed mixing the two vectors in 1:1 ratio in OptiMEM and using Lipofectamine 2000 as transfection reagent. After 8 h, transfection medium was replaced by culture medium and, after additional 24 h, cells were gently washed with PBS and fixed with 3.8% paraformaldehyde (PFA) in PBS for 15 min. To confirm the mitochondrial localization of Venus fluorescence signal, an immunostaining with the primary antibody anti-MTND1 (Complex I) and secondary fluorescent antibody anti-mouse Alexa Fluor^TM^ 633 (Table S2, Supplementary material) was performed. Images were acquired by means of Leica SP5 confocal microscope at 40X magnification at the Imaging facility of the Department of Biology, University of Padova (Italy), and ultimately processed with ImageJ Fiji software, quantifying the corrected total fluorescence per cell of Venus signal.

### Measurement of respiratory chain complexes activities

To prompt metabolic switching from glycolysis to respiration before respiratory chain activity assessment, fibroblasts were grown for 48 h in DMEM no glucose, supplemented with 2 mM D-Glucose, 5% (v/v) FBS, 2 mM L-glutamine and 100 µg/mL penicillin at 37 °C and 5% CO_2_. The enzymatic activity of each complex belonging to the electron transport chain (ETC) was measured according to the protocol described by Spinazzi et al. [73]. Briefly, 50 × 10^6^ cells from each line were harvested, sedimented, washed twice with PBS, and prepared to obtain the mitochondrial-enriched fraction. Each sample protein content was then quantified according to the BCA method (Thermo Fisher Scientific) and the enzymatic activities were measured by means of a single-wavelength multicuvette spectrophotometer (Varian Cary UV-Vis 100) at 37 °C. Activities of the mitochondrial OXPHOS complexes were normalized to protein content and citrate synthase activity.

### Cellular respiration measurements

Oxygen consumption rate (OCR) was measured by means of a Seahorse XFe24 Analyzer (Agilent technologies). For each cell line, 30 × 10^3^ cells/well were seeded in a 24-well Seahorse plate. The day after seeding, culture medium was replaced by assay medium composed of DMEM (Sigma, D5030), 1 mM sodium pyruvate (GIBCO Life Technologies), 4 mM L-glutamine (GIBCO Life Technologies), 25 mM glucose (Sigma-Aldrich) and 1% (v/v) FBS (GIBCO Life Technologies), pH 7.4. OCR was measured at fixed time points under basal conditions and after the addition of 2.5 μM oligomycin (Sigma-Aldrich), 1.5 μM FCCP (Sigma-Aldrich), 1 μM rotenone (Sigma-Aldrich) and 1 μM antimycin A (Sigma-Aldrich). At the end of each experiment, cellular respiration rates were normalized to the total protein concentration as assessed by BCA assay after cell lysis. Bioenergetic parameters were obtained as described in [74].

### Transmission Electron Microscopy (TEM)

Mitochondrial morphology and ultrastructure were analyzed by means of TEM at the electron microscopy facility of the Department of Biology, University of Padova (Italy). For the preparation of samples, 40 × 10^3^ cells/well were seeded in a 24-well plate to reach approximately 80% final confluence. The day after seeding, cells were fixed at 4 °C for 1 h and 30 min in 2.5% glutaraldehyde (Electron Microscopy Sciences) in 0.1 M sodium cacodylate buffer pH 7.4. Samples were then post-fixed with 1% osmium tetroxide/1% potassium ferrocyanide in 0.1 M sodium cacodylate buffer pH 7.4 for 1 h at 4 °C. After three buffer washes, samples were dehydrated in a graded ethanol series and embedded in an epoxy resin (Sigma-Aldrich). Ultrathin sections (60–70 nm) were obtained with a Leica EM UC7 ultramicrotome, counterstained with uranyl acetate and lead citrate and then examined under a Tecnai G2 (FEI) transmission electron microscope operating at 120 kV. Images were captured with a Veleta (Olympus Soft Imaging System) digital camera. Morphometric analysis of mitochondrial ultrastructure was performed using ImageJ Fiji software, as described in [75]. Briefly, for each mitochondrion, the mitochondria length, considered as the longest distance between any two points of the organelle, and the ratio between the number of cristae junctions and the number of cristae, were analyzed.

### Prussian blue staining

For the detection of cellular iron deposits by Prussian blue staining, 250 × 10^3^ fibroblasts/well were seeded on 25 mm diameter cover glasses in 6-well plate. The following day, cells were gently washed twice with PBS and fixed with ice-cold 100% methanol for 10 min at −20 °C. After two washes with milliQ water, cells were incubated for 30 min at room temperature with a freshly prepared staining solution composed of equal parts of 20% (w/v) hydrochloric acid and 10% (w/v) potassium ferrocyanide solutions. After three rinses with milliQ water, cover slides were dried and mounted with mowiol mounting medium (Sigma-Aldrich). Images were acquired by means of Leica DM6 B microscope at 100X magnification at the Imaging facility of the Department of Biology, University of Padova (Italy).

### Inductively Coupled Plasma Mass Spectrometry (ICP-MS)

Inductively Coupled Plasma Mass Spectrometry (ICP-MS) was employed to quantify the total intracellular iron content. For each cell line, 45 × 10⁶ cells were collected, sedimented, and washed twice with ice-cold PBS. Cell pellets were digested by adding 0.6 g of 65% HNO₃ (Fluka; 84378) and heating in a boiling water bath (100 °C) for 2 h. The resulting solutions were diluted with Milli-Q water and filtered through 0.45 μm syringe filters into clean tubes prior to ICP-MS analysis. Measurements were performed using an ICP-MS system (7700x ICP-MS, Agilent Technologies International Japan, Ltd., Tokyo, Japan) equipped with an octapole collision cell operating in kinetic energy discrimination mode. Operating conditions and data acquisition parameters were adapted from a validated protocol [76].

### Measurements of mitochondrial iron and Reactive Oxygen Species (ROS)

Mitochondrial labile iron levels were evaluated using Mito-FerroGreen (MFG) (Dojindo). Mitochondrial ROS were measured using MitoSOX™ Red (Thermo Fisher Scientific). For the experiments, 5 × 10^3^ cells/well were seeded in a 96-well TC-Treated Black μCLEAR plate (Greiner Bio-One). The day after seeding, cells were stained with MFG or MitoSOX according to the manufacturer’s recommendations. Briefly, cells were washed three times with Hanks’ Balanced Salt Solution (HBSS) (GIBCO Life Technologies) and incubated in 5 μM MFG or 2.5 μM MitoSOX Red solution for 30 min, under 5% CO_2_ at 37 °C, in the dark. Nuclei were counter-stained with Hoechst (Thermo Fisher Scientific, 33342; 1:2000) and to confirm the localization of MFG or oxidized MitoSOX fluorescence signal within mitochondria, 100 nM MitoTracker™ Red (Thermo Fisher Scientific) or MitoTracker™ Green (Thermo Fisher Scientific) was added to the staining solution. After three washes in HBSS, live imaging fields were acquired using the Operetta CLS™ High-Content Analysis System (Revvity) at the HiTS facility Imaging facility of the Department of Biology, University of Padova (Italy), using a 40X Air/0.75 objective and in 6 Z-stack planes at 0.5 µm Z step size. Excitation and emission wavelength ranges used for the measurement of MFG and oxidized MitoSOX Red were 490–515 nm/525–580 nm and 530–560 nm/570–650 nm, respectively. MitoTracker Red, MitoTracker Green and Hoechst signals were acquired using 530–560 nm, 460–490 nm, 355–385 nm as excitation wavelengths interval and 570–650 nm, 500–550 nm, 430–500 nm as emission wavelengths intervals, respectively. To measure the intensity of MFG or oxidized MitoSOX Red fluorescent signal colocalizing within mitochondria, the images were analyzed by means of Harmony® High-Content Imaging and Analysis Software with PhenoLOGIC™ algorithm (Revvity).

### Morphological analysis of mitochondria upon H_2_O_2_ treatment

To evaluate the cellular oxidative stress response induced by hydrogen peroxide, 30 × 10^3^ fibroblasts/well were seeded on 13 mm diameter cover glasses in 24-well plate the day before the treatments. Cells were then washed three times with HBSS and incubated with 25 or 100 μM H_2_O_2_ solutions in HBSS for 2 h, under 5% CO_2_ at 37 °C. At the end of the treatments, cells were fixed with 3.8% PFA and immunostained using anti-MTND1 (Complex I) and anti-mouse Alexa Fluor^TM^568 as primary and secondary antibodies, respectively (Table S2, Supplementary material). Images were acquired by means of Zeiss LSM 900 confocal microscope at 63X magnification and ZEN Software at the Imaging facility of the Department of Biology, University of Padova, and analyzed with ImageJ Fiji software. Mitochondrial morphology changes in response to H_2_O_2_-induced stress were assessed by measuring the mitochondrial aspect ratio (i.e., the ratio between the long axis over the short axis of an ellipse equivalent to the mitochondrion) for all mitochondria belonging to each analyzed cell.

### Protein expression and purification

DNA sequences coding for wild type FDX2 (residues 53-183), mutant FDX2 (residues 51-183) and FDX2^66-183^ (residues 66-183), fused in frame at the N-terminus to a 6xHis-tag sequence and a following TEV-cleavage site, were cloned between NdeI and XhoI restriction sites of pET29b(+) and used to transform *E. coli* BL21 (DE3) cells. All proteins were expressed and purified by following the protocol previously reported by us for FDX2^66-183^ [21]. The final protein samples were exchanged to 30 mM HEPES, 150 mM NaCl, pH 7.5, and stored at −80 °C.

### Absorption electronic and NMR Spectroscopies

UV/visible absorption (250–800 nm) and UV/visible-CD (300–750 nm) spectra were recorded at 298 K using a Cary 50 Eclipse spectrophotometer and a JASCO J-810 spectropolarimeter, respectively. The purified protein samples were in 50 mM phosphate buffer, 150 mM NaCl at pH 7.0 or in 30 mM HEPES buffer, 150 mM NaCl pH 7.5 in a 1 cm path length sealed cuvette. All spectroscopy data shown are representative of three or more independent experiments.

1D ^1^H paramagnetic NMR spectra were recorded at 298 K for each cluster-oxidized sample and on the same samples treated with 10 mM dithionite. All spectra were recorded on a Bruker AV400 MHz spectrometer, equipped with a 5-mm ^1^H selective high-power probe without gradients, following standard NMR pulse sequences and data processing [77].

Backbone resonance assignment of [2Fe-2S]^2+^ FDX2^66-183^ was already available [21]. Solution NMR experiments for backbone resonance assignment were performed on ^13^C and ^15^N labeled [2Fe-2S]^2+^ mutant FDX2 and [2Fe-2S]^2+^ wild type FDX2 samples, in a buffer solution containing 30 mM HEPES, 150 mM NaCl at pH 7.5. Standard 3D triple resonance experiments were acquired at 700 MHz spectrometer at 298 K. 3D NMR spectra were processed using the standard Bruker software and analyzed with CARA program. The backbone NMR chemical shifts (δ^15^N, δ^13^C′, δ^13^C^α^, δ^13^C^β^, δ^1^H^α^, and δ^1^H^N^) of cluster-oxidized proteins were used to perform secondary structure analysis by TALOS-N [78]. NMR experiments for measuring the ^15^N longitudinal (R_1_) and transverse (R_2_) relaxation rates and {^1^H}^15^N heteronuclear NOE values were recorded at 298 K on Bruker Avance spectrometers operating at 500 MHz. ^15^N R_1_, ^15^N R_2_, and steady-state {^1^H}^15^N heteronuclear NOEs were measured with previously described pulse sequences [79]. ^15^N R_2_ were measured with a refocusing time (τ_CPMG_) of 450 μs with the Carr-Purcell-Meiboom-Gill (CPMG) sequence [80].

### Statistical analysis

All numerical data, analyzed by GraphPad Prism, are expressed as mean ± SEM, unless otherwise stated. Statistical analysis and significance were performed and assessed as specified in the figure legends, with *p* ≤ 0.05 accepted as statistically significant.

## Supplementary information

Complete supplementary files

Original WB

## Data Availability

All data generated and analyzed in this study are included in this published article and its supplementary information files.
