# Supplementary material for: A novel mutation in FDX2 provides insights into the pathogenesis of MEOAL mitochondrial neuromuscular disease
Source: Cell Death Dis. 2025 Dec 10;17(1):59. doi: 10.1038/s41419-025-08323-3 (PMC12824309; doi:10.1038/s41419-025-08323-3)
Supplement: Supplementary file 1 — Complete supplementary files [file 41419_2025_8323_MOESM1_ESM.pdf]

Recombinant wild type FDX2

AGEEDAGGPERPGDVVNVVFVDRSGQRIPVSGRVGDNVLHLAQRHGVDLEGACEASL
ACSTCHVYVSEDHLDLLPPPEEREDDMLDMAPLLQENSRLGCQIVLTPELEGAEFTL
PKITRNFYVDGHVPKPH

Recombinant mutant FDX2

ALSPSSSSAFALPSVPRVNVVFVDRSGQRIPVSGRVGDNVLHLAQRHGVDLEGACEA
SLACSTCHVYVSEDHLDLLPPPEEREDDMLDMAPLLQENSRLGCQIVLTPELEGAEF
TLPKITRNFYVDGHVPKPH

Recombinant FDX2$^{66\text{-}183}$

DVVNVVFVDRSGQRIPVSGRVGDNVLHLAQRHGVDLEGACEASLACSTCHVYVSEDH
LDLLPPPEEREDDMLDMAPLLQENSRLGCQIVLTPELEGAEFTLPKITRNFYVDGHV
PKPH

Color code:

- Exon 2
- Exon 3
- Exon 4
- Exon 5
- Intron2 coded polypeptide

SUPPLEMENTARY FIGURE 1

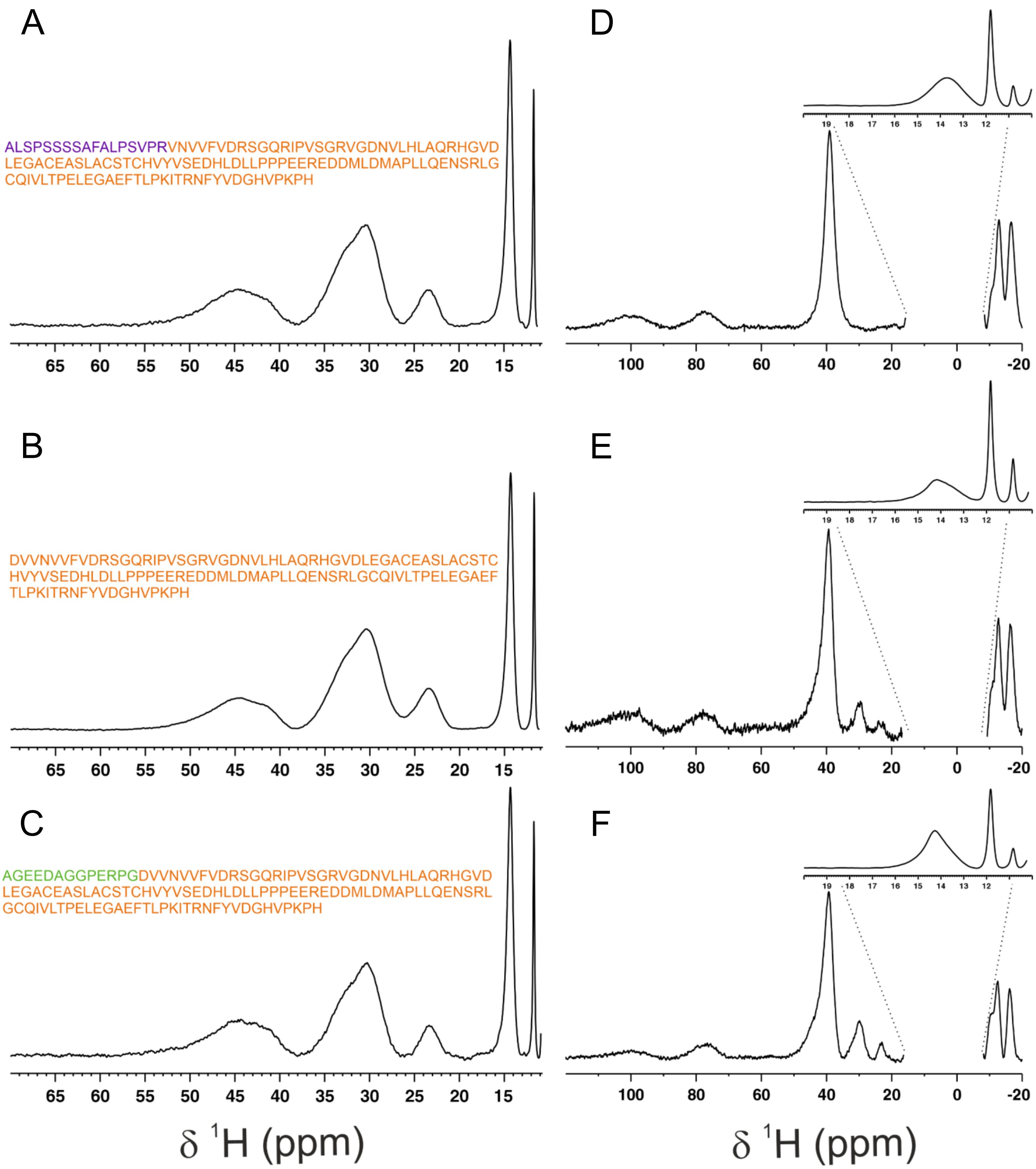

SUPPLEMENTARY FIGURE 2

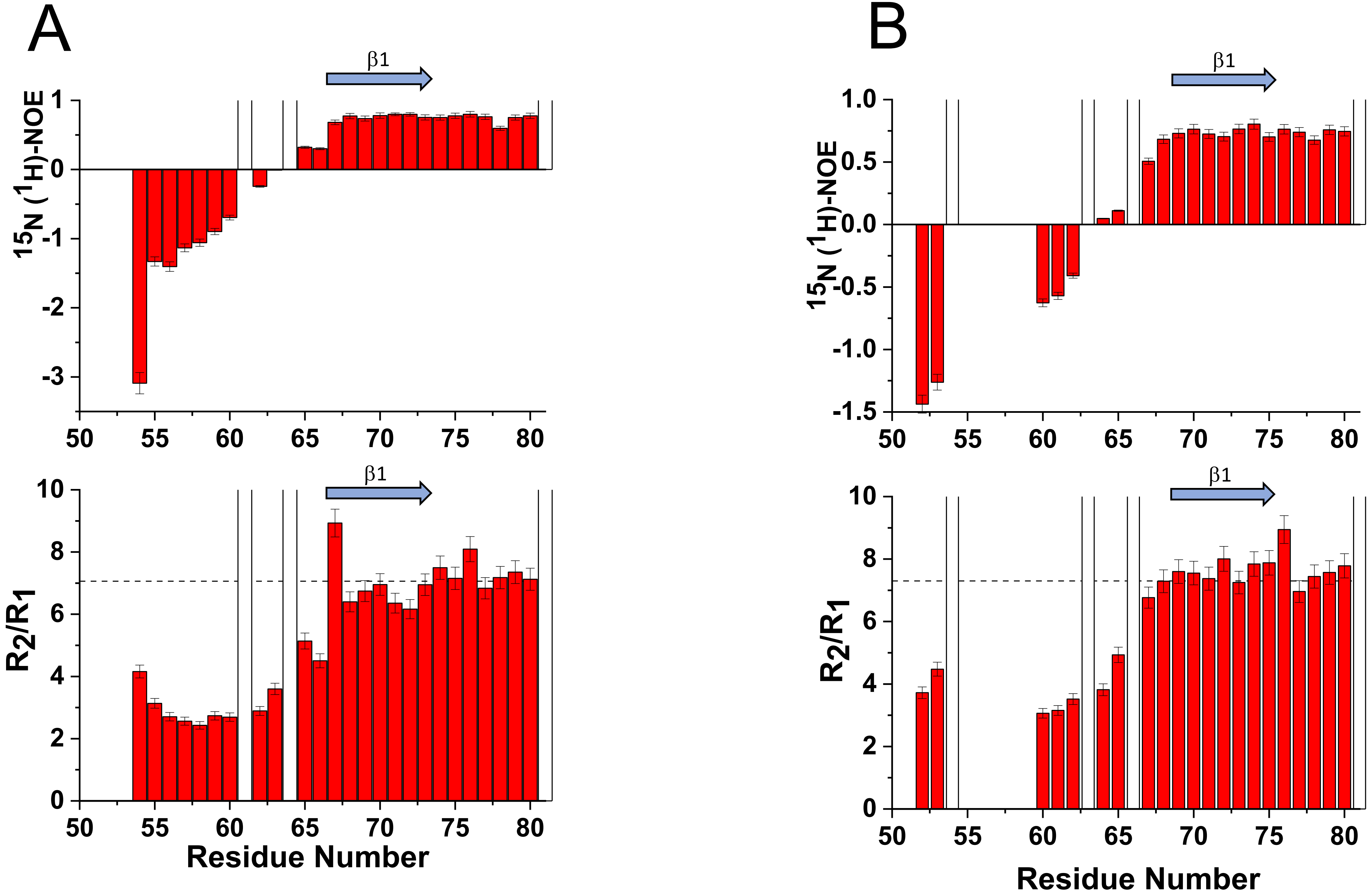

SUPPLEMENTARY FIGURE 3

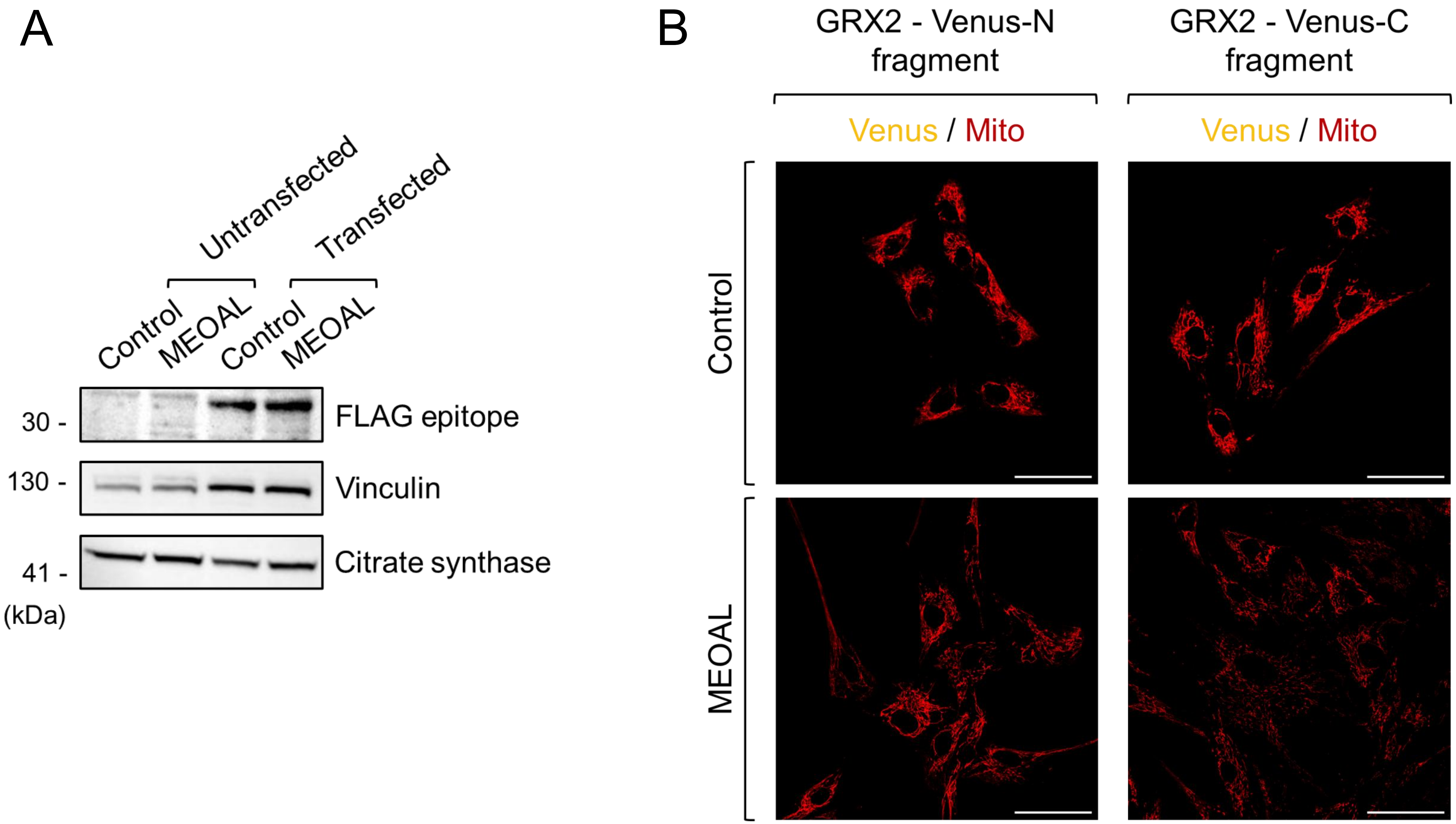

SUPPLEMENTARY FIGURE 4

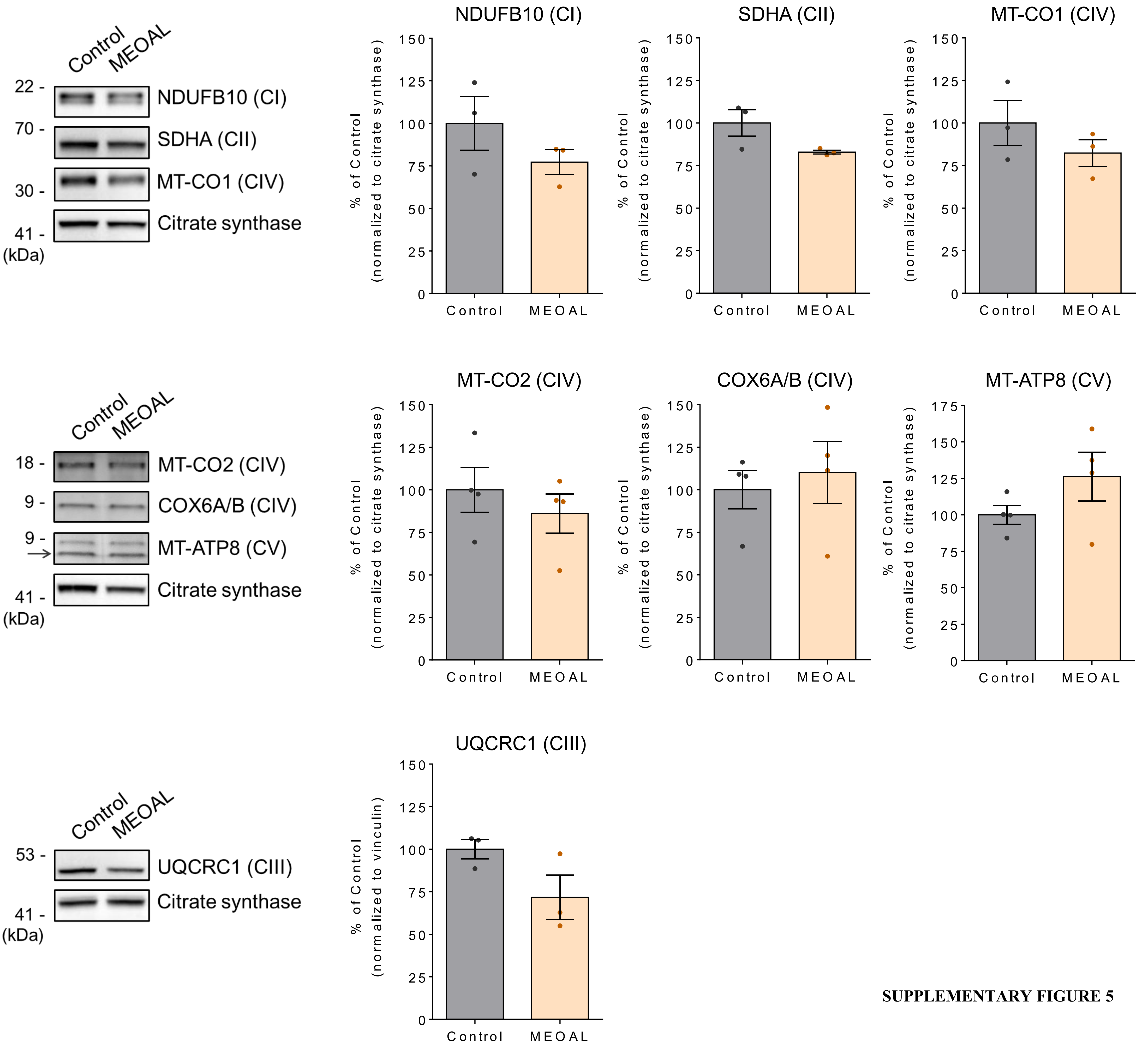

SUPPLEMENTARY FIGURE 5

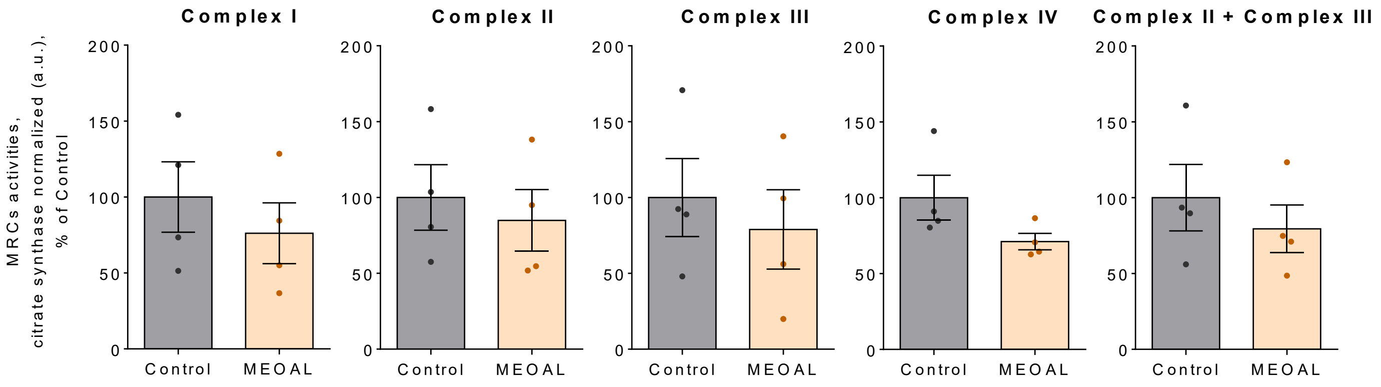

SUPPLEMENTARY FIGURE 6

SUPPLEMENTARY FIGURE CAPTIONS

Supplementary Fig. 1. Amino acid sequence of the recombinant FDX2 proteins analyzed in this work. Color code is reported in the figure.

Supplementary Fig. 2. Comparison of the 1D ^{1}H paramagnetic NMR spectra of the three produced recombinant proteins of [2Fe-2S] FDX2. Mutant FDX2 (**A, D**), FDX2$^{66\text{-}183}$ (**B, E**) and wild type FDX2 (**C, F**). The NMR spectra were recorded at 298 K on a Bruker AV400 MHz spectrometer on the oxidized +2 (left, A, B, and C) and reduced +1 (left, D, E, and F) cluster redox states. Buffer conditions: 30 mM HEPES, 150 mM NaCl at pH 7.5. Amino acid sequences of the three FDX2 recombinant proteins are also reported showing by color-code the different N-terminal segments of wild type and mutant FDX2.

Supplementary Figure 3. Monitoring the backbone dynamics of the N-terminal segment of [2Fe-2S]$^{2+}$ mutant and wild type FDX2 proteins. ^{1}H(^{15}N) NOE values and ratios of the transversal (R_2) vs. longitudinal (R_1) relaxation rates measured at 500 MHz and 298 K for the N-terminal residues of [2Fe-2S]$^{2+}$ wild type FDX2 (**A**) and [2Fe-2S]$^{2+}$ mutant FDX2 (**B**). Proline residues are indicated as white bars. The values for residues 55-59 in [2Fe-2S]$^{2+}$ mutant FDX2 are not present as their backbone NHs are not assigned. The backbone dynamics of both N-terminal segments in [2Fe-2S]$^{2+}$ mutant FDX2 and [2Fe-2S]$^{2+}$ wild type FDX2 are characterized by fast ps-ns fluctuations as monitored by R_2/R_1 values below the mean and by negative or very low {^{1}H}^{15}N NOE values.

Supplementary Fig. 4. A) Immunoblot analysis of GRX2-Venus transfection efficiency in healthy control and MEOAL cells. The expression levels of the FLAG-tagged GRX2 – Venus-N fusion protein were analyzed in whole cell extract from healthy control and MEOAL fibroblasts transiently co-transfected or not with the plasmid vectors used in the Fe-S cluster Fluorescent Assay. The same amount of protein lysate (*i.e.* 70 μg) was loaded in each lane. For the immuno-detection of the protein, an anti-FLAG tag primary antibody was used. Vinculin and citrate synthase were used as loading controls. A representative image of one of three independent experiments is reported. **B)**

Negative control of the Fe-S cluster Fluorescent Assay. Representative confocal images of healthy control and MEOAL cells individually transfected with either of the two plasmid vectors encoding the Venus fragments fused to GRX2. Venus fluorescent signal (yellow) was absent. Mitochondria (red) were immunostained using a primary antibody against respiratory complex I. Scale bar: 60 μm.

Supplementary Fig. 5. Comparative analysis of respiratory complexes subunits in healthy control and MEOAL cells. Western blotting analysis of respiratory chain complexes subunits not containing Fe-S clusters in whole cells extracts from healthy control and MEOAL patient fibroblasts. Equal amounts of protein (i.e. 40 μg) were loaded in each lane. Citrate synthase was used as loading control. Protein levels were quantified after normalization with citrate synthase and expressed as a percentage of control levels. Reported data result from the mean of least three independent experiments ± SEM. Statistical significance was determined using unpaired t-test (non statistically significant, compared to control).

Supplementary Fig. 6. Complex I-IV individual activities. Activities of respiratory complexes I-IV and complexes II + III (nmol / min · mg of protein) were measured in healthy and MEOAL cells, normalized to that of citrate synthase and expressed as percentage of control activity. Reported data result from the mean of four independent experiments ± SEM and statistical significance was determined using unpaired t-test (non statistically significant, compared to control).

Table S1. Molecular, biochemical and clinical characterization of the reported cases of MEOAL

<table>
<tr><th>Reported clinical case</th><th>FDX2 variant</th><th>Protein mutation</th><th>Age of onset</th><th>Clinical features</th></tr>
<tr><td>Spiegel et al. 2014</td><td>Homozygous c.1A>T</td><td>p.M1L</td><td>15 years</td><td>Muscle weakness with exercise intolerance; recurrent severe episodes of myoglobinuria and rhabdomyolysis</td></tr>
<tr><td rowspan="6">Gurgel-Giannetti et al. 2018</td><td rowspan="6">Homozygous c.431C>T</td><td rowspan="6">p.P144L</td><td>6 months</td><td rowspan="6">Optic atrophy, myopathy, recurrent cramps, myalgia and muscle weakness; reversible/partially reversible leukoencephalopathy on MRI</td></tr>
<tr><td>1 year and 4 months</td></tr>
<tr><td>1 year</td></tr>
<tr><td>1 year</td></tr>
<tr><td>8 months</td></tr>
<tr><td>6 months</td></tr>
<tr><td>Lebigot et al. 2017 and Montealegre et al. 2022</td><td>Homozygous c.1A>T</td><td>p.M1L</td><td>5 years</td><td>Myopathy with acute episodes of rhabdomyolysis and lactic acidosis; generalized weakness and myalgia</td></tr>
<tr><td>Aggarwal et al. 2021</td><td>Homozygous c.12G>T</td><td>p.M4I</td><td>10 years</td><td>Myopathy with recurrent rhabdomyolysis and lactic acidosis; significant exercise intolerance and motor impairment; no optic atrophy or leukoencephalopathy</td></tr>
<tr><td>Gkiourtzis et al. 2023</td><td>Homozygous c. 10A>T</td><td>p.M4L</td><td>13 years and 5 months</td><td>Myopathy and muscle weakness with exercise intolerance and recurrent episodes of rhabdomyolysis, lactic acidosis and myoglobinuria</td></tr>
<tr><td>Wongkittichote et al. 2024</td><td>Compound heterozygous c.1A>T; c.146-2A>G</td><td>p.M1L
Abnormal splicing</td><td>6 years</td><td>Progressive myopathy with acute episodes of rhabdomyolysis affecting respiratory function; lactic acidosis and ketosis</td></tr>
<tr><td>Current report</td><td>Homozygous c. 200+4A>G</td><td>Abnormal splicing</td><td>9 months</td><td>Ataxia, muscle weakness, cerebellar and foveal hypoplasia, mild lactic acidosis; progressive motor fatigue and gain weight</td></tr>
</table>

Table S2. List of antibodies used in this work

	Antibody	*Supplier*	*Condition of use*
WB	Anti – FDX2	Affinity purified, Sheftel AD *et al.* 2010 [15]	1:250 in TBS T 1X – O/N, 4°C
	Anti – Citrate synthase	Thermo Fisher Scientific; MA5-17264	1:1000 in TBS T 1X – O/N, 4°C
	Anti-HA Tag	Thermo Fisher Scientific; 26183	1:1000 in TBS T 1X – O/N, 4°C
	Anti – Vinculin	Sigma-Aldrich; V9264	1:1000 in TBS T 1X – O/N, 4°C
	Anti – NDUFS1	Santa Cruz; SC-271510	1:500 in TBS T 1X – O/N, 4°C
	Anti – NDUFS8	Santa Cruz; SC-515527	1:500 in TBS T 1X – O/N, 4°C
	Anti – NDUFS7	Thermo Fisher Scientific; PA5-106367	1:500 in TBS T 1X – O/N, 4°C
	Anti – NDUFV1	Thermo Fisher Scientific; PA5-21426	1:500 in TBS T 1X – O/N, 4°C
	Anti – NDUFV2	Santa Cruz; SC-271620	1:500 in TBS T 1X – O/N, 4°C
	Anti – SDHB	Santa Cruz; SC-59688	1:500 in TBS T 1X – O/N, 4°C
	Anti – UQCRFS1	Santa Cruz; SC-271609	1:500 in TBS T 1X – O/N, 4°C
	Anti- FECH	Santa Cruz; SC-377377	1:500 in TBS T 1X – O/N, 4°C
	Anti-ACO2	Thermo Fisher Scientific; MA1-029	1:1000 in TBS T 1X – O/N, 4°C
	Anti-PDH-E2	Cell Signaling Technology (CST); 12362S	1:2500 in 0.5% BSA, TBS T 1X – 90 min, RT
	Anti-Lipoyl PDH-E2	Calbiochem/Merck; 437692	1:1000 in 0.5% BSA, TBS T 1X – 90 min, RT
	Anti-NDUFB10	Abcam; AB196019	1:800 in TBS T 1X – O/N, 4°C
	Anti-SDHA	Santa Cruz; SC-166947	1:800 in TBS T 1X – O/N, 4°C
	Anti-UQCRC1	Santa Cruz; SC-65238	1:500 in TBS T 1X – O/N, 4°C
	Anti-MT-CO1	Abcam; AB14705	1:800 in TBS T 1X – O/N, 4°C
	Anti-MT-CO2	H. Schägger, I. Wittig (Frankfurt, Germany)	1:2000 in 0.5% BSA, TBS T 1X – 90 min, RT
	Anti-COX6A/B	H. Schägger, I. Wittig (Frankfurt, Germany)	1:4000 in 0.5% BSA, TBS T 1X – 90 min, RT
	Anti-MT-ATP8	Protein Tech Group (PTG); 26723-1-AP	1:800 in 0.5% BSA, TBS T 1X – 90 min, RT
	Anti-ACO1 (IRP1)	Proteintech; 12406-1-AP	1:1000 in TBS T 1X – O/N, 4°C
	Anti-IREB2 (IRP2)	Thermo Fisher Scientific; PA1-16544	1:1000 in TBS T 1X – O/N, 4°C
	Anti - Ferritin	Thermo Fisher Scientific; MA5-32244	1:1000 in TBS T 1X – O/N, 4°C
	Anti - TfR	Thermo Fisher Scientific; 13-6800	1:1000 in TBS T 1X – O/N, 4°C
	Anti-FtMt	Abcam; 124889	1:500 in TBS T 1X – O/N, 4°C
	Anti –Catalase	Abcam; AB1877	1:1000 in 5% BSA, TBS T 1X – O/N, 4°C
	Anti –GLRX2	Proteintech; 13381-1-AP	1:1000 in TBS T 1X – O/N, 4°C
	Anti –SOD1	Sigma-Aldrich; HPA001401	1:1000 in TBS T 1X – O/N, 4°C
	Anti –SOD2	Thermo Fisher Scientific; MA1-106	1:1000 in TBS T 1X – O/N, 4°C
	Anti –FLAG Tag	Thermo Fisher Scientific; 740001	1:500 in TBS T 1X – O/N, 4°C
IF	Anti-MTND1	Thermo Fisher Scientific; 43-8800	1:100 in 1% w/v BSA and 0.1% Tween-20 in PBS – O/N, 4°C
	Anti-mouse Alexa Fluor^{TM} 633 (FeS cluster fluorescent assay)	Thermo Fisher Scientific, A-21050	1:100 in 1% w/v BSA and 0.1% Tween-20 in PBS – 1 hour, RT
	Anti-mouse Alexa Fluor^{TM} 568	Thermo Fisher Scientific, A-11031	1:100 in 1% w/v BSA and 0.1% Tween-20 in PBS – 1 hour, RT
