# Supplementary material for: A novel mutation in FDX2 provides insights into the pathogenesis of MEOAL mitochondrial neuromuscular disease
Source: Cell Death Dis. 2025 Dec 10;17(1):59. doi: 10.1038/s41419-025-08323-3 (PMC12824309; doi:10.1038/s41419-025-08323-3)

Figure 4

Panel A

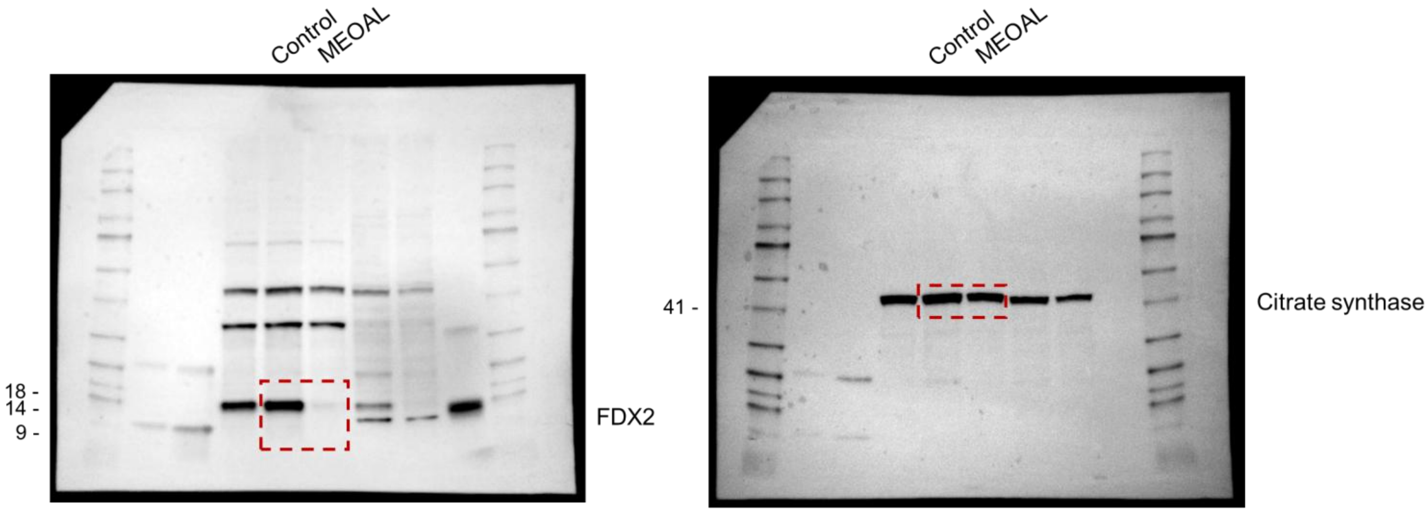

Panel B

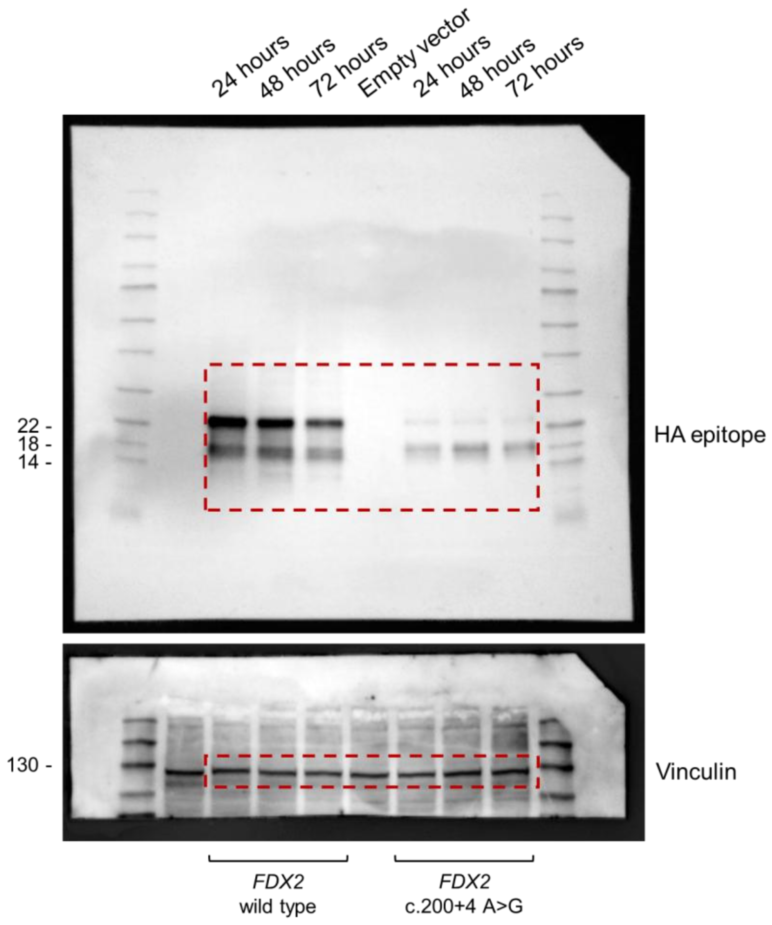

Figure 5

Panel B

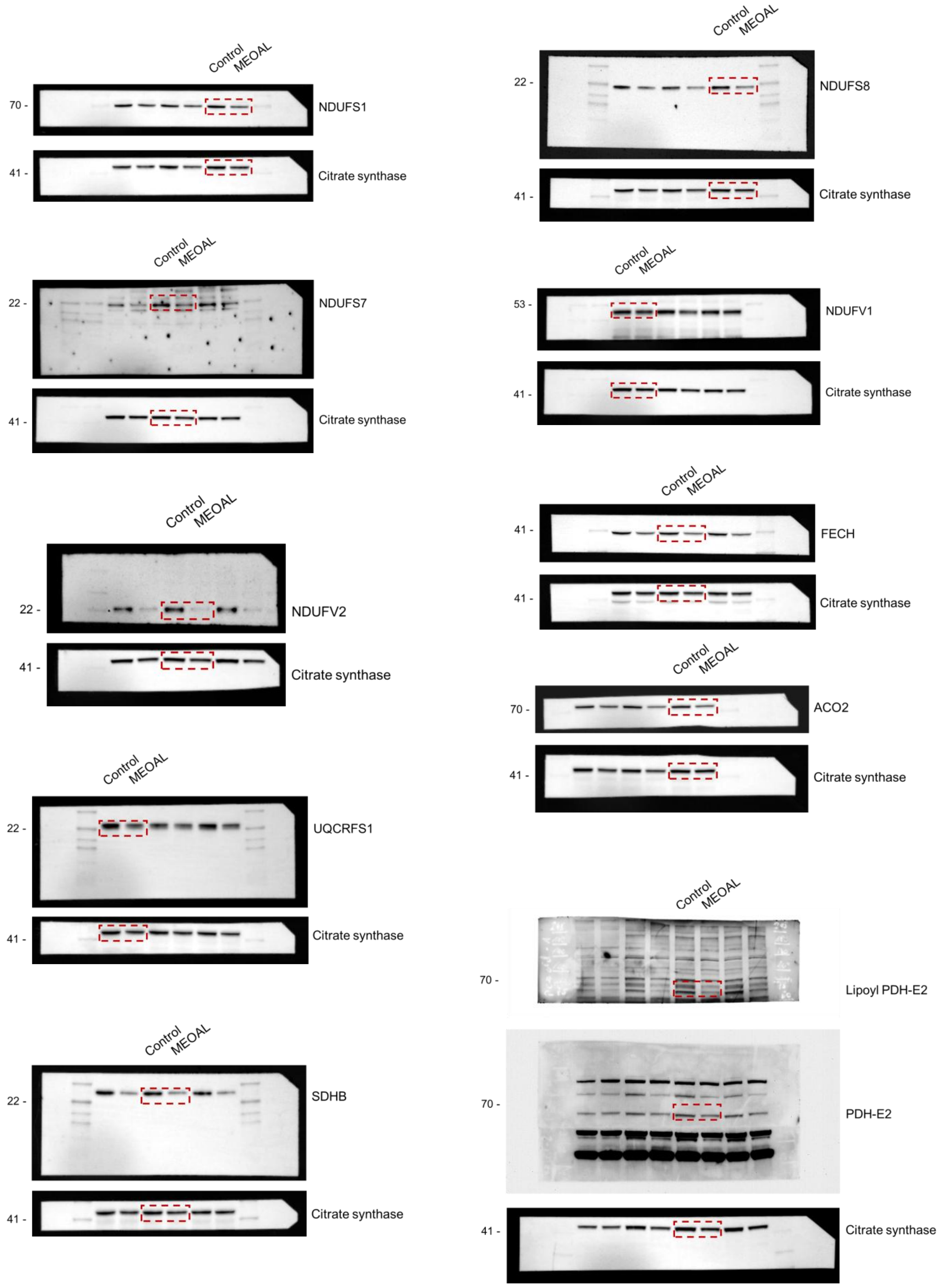

Figure 7

Panel D

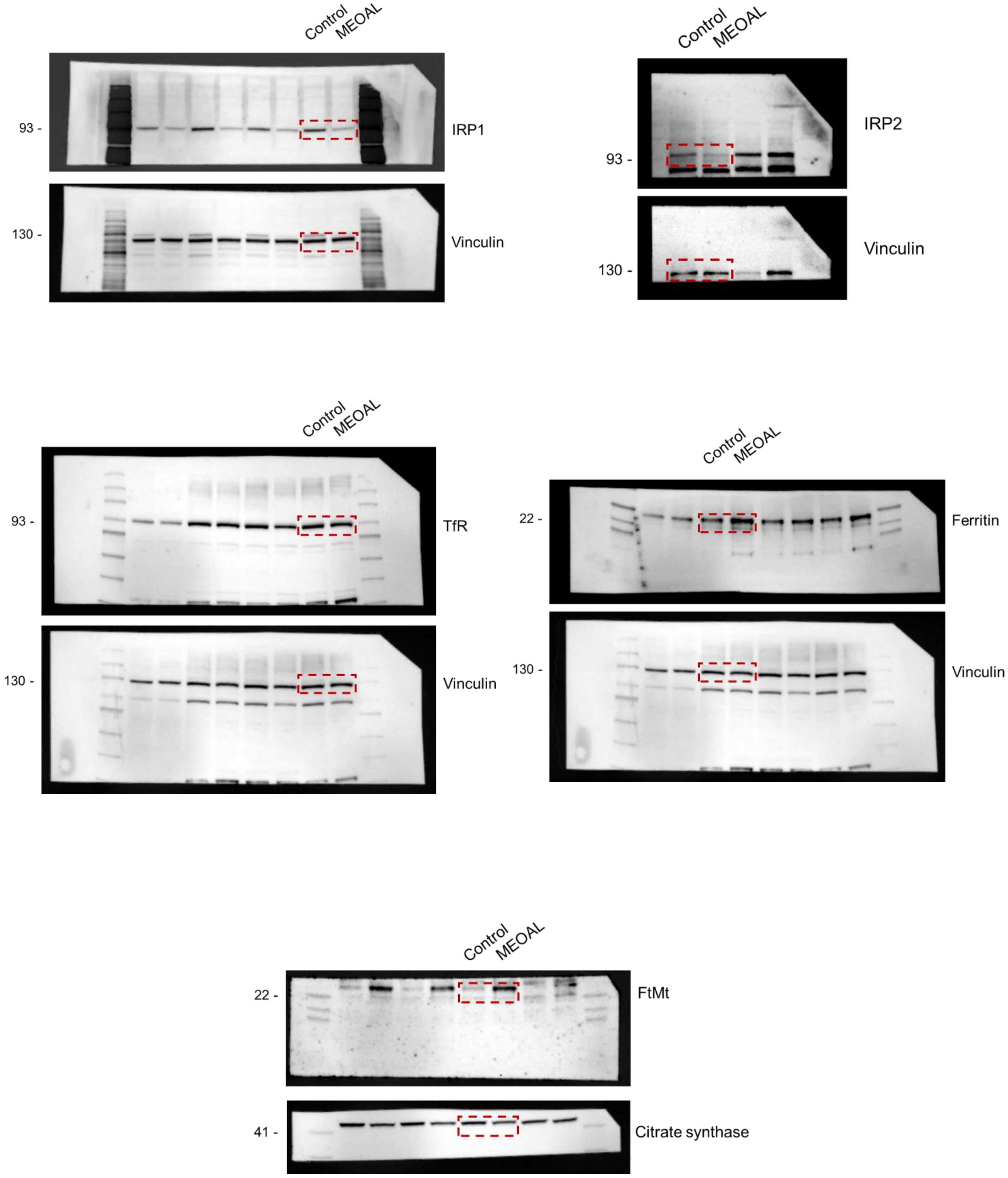

Figure 8

Panel C

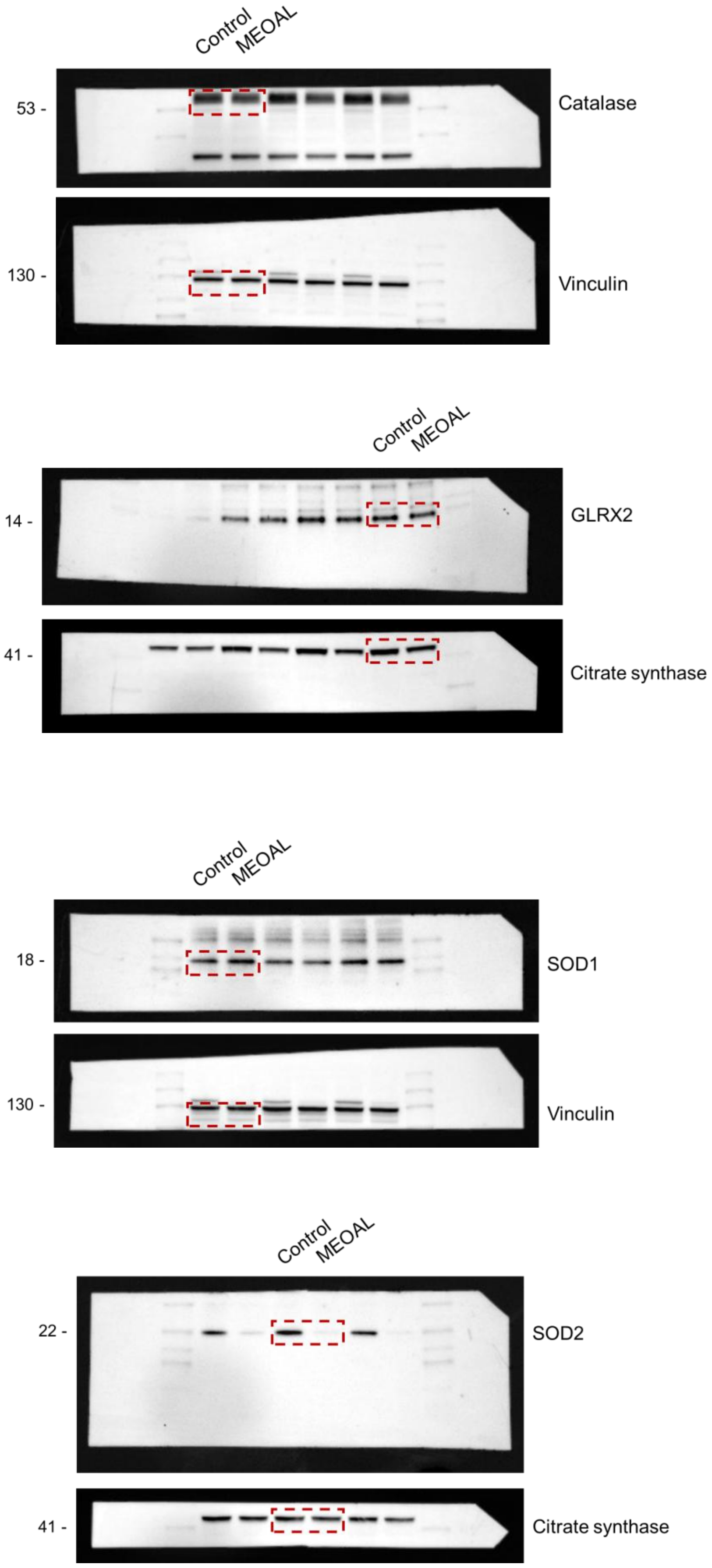

Supplementary Figure 4

Panel A

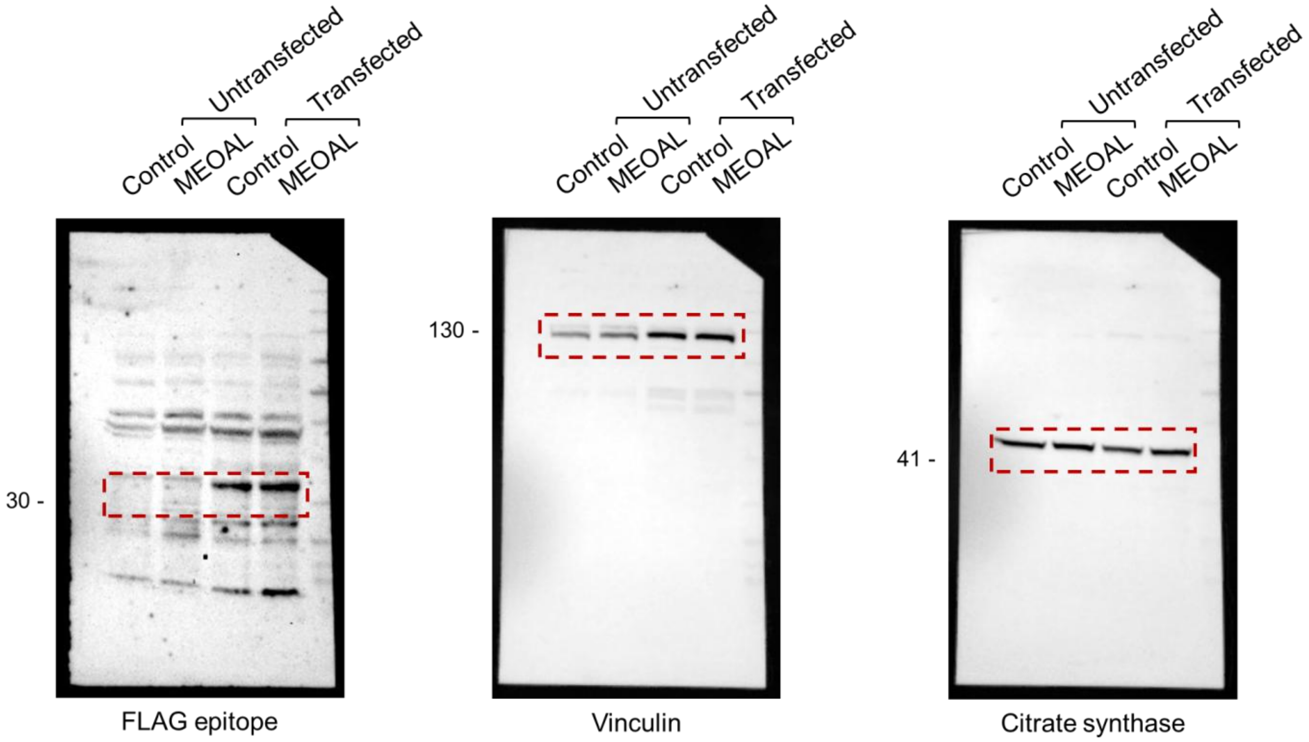

Supplementary Figure 5

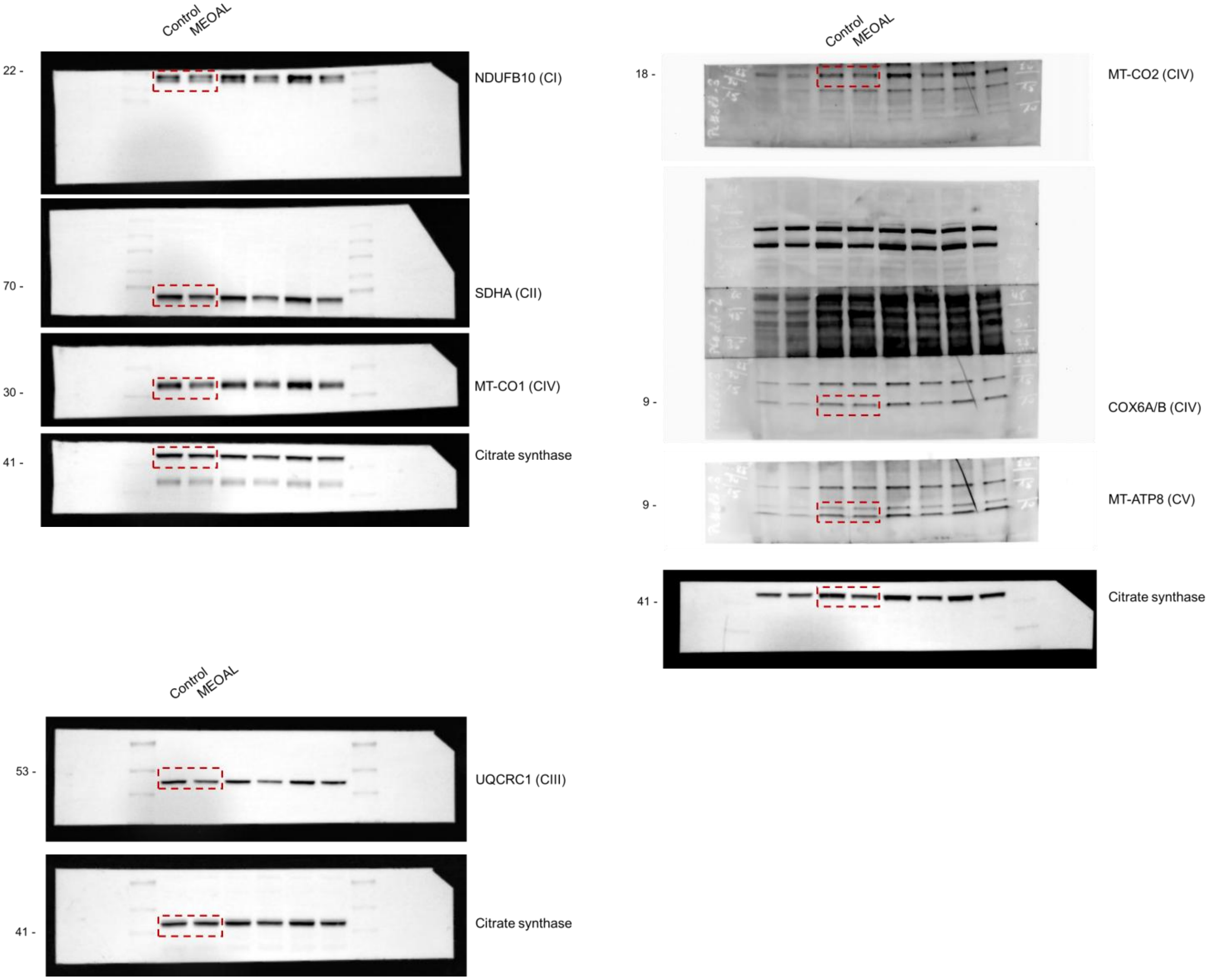

Supplement: Supplementary file 2 — Original WB [file 41419_2025_8323_MOESM2_ESM.pdf]
